# Identifying long-term survivors and those at higher or lower risk of relapse among patients with cytogenetically normal acute myeloid leukemia using a high-dimensional mixture cure model

**DOI:** 10.1186/s13045-024-01553-6

**Published:** 2024-05-03

**Authors:** Kellie J. Archer, Han Fu, Krzysztof Mrózek, Deedra Nicolet, Alice S. Mims, Geoffrey L. Uy, Wendy Stock, John C. Byrd, Wolfgang Hiddemann, Jan Braess, Karsten Spiekermann, Klaus H. Metzeler, Tobias Herold, Ann-Kathrin Eisfeld

**Affiliations:** 1 Division of Biostatistics, College of Public Health, The Ohio State University, 240 Cunz Hall, 1841 Neil Avenue, Columbus, OH 43210 USA; 2 Google, Inc., Mountain View, CA USA; 3 Clara D. Bloomfield Center for Leukemia Outcomes Research, The Ohio State University Comprehensive Cancer Center, Columbus, OH USA; 4 Alliance Statistics and Data Management Center, The Ohio State University Comprehensive Cancer Center, Columbus, OH USA; 5 Department of Medicine, Division of Oncology, Washington University School of Medicine, St. Louis, MO USA; 6 Department of Medicine, Section of Hematology/Oncology, University of Chicago, Chicago, IL USA; 7 Department of Internal Medicine, University of Cincinnati, Cincinnati, OH USA; 8 Laboratory for Leukemia Diagnostics, Department of Medicine III, University Hospital, LMU Munich, Munich, Germany; 9 Department of Oncology and Hematology, Hospital Barmherzige Brüder, Regensburg, Germany; 10 Department of Hematology and Cellular Therapy, University Hospital Leipzig, Leipzig, Germany

**Keywords:** Prognostic classification, Penalized survival model, Regularized survival model, Least absolute shrinkage and selection operator, LASSO, Cox proportional hazards

## Abstract

Patients with cytogenetically normal acute myeloid leukemia (CN-AML) may harbor prognostically relevant gene mutations and thus be categorized into one of the three 2022 European LeukemiaNet (ELN) genetic-risk groups. Nevertheless, there remains heterogeneity with respect to relapse-free survival (RFS) within these genetic-risk groups. Our training set included 306 adults on Alliance for Clinical Trials in Oncology studies with de novo CN-AML aged < 60 years who achieved a complete remission and for whom centrally reviewed cytogenetics, RNA-sequencing, and gene mutation data from diagnostic samples were available (Alliance trial A152010). To overcome deficiencies of the Cox proportional hazards model when long-term survivors are present, we developed a penalized semi-parametric mixture cure model (MCM) to predict RFS where RNA-sequencing data comprised the predictor space. To validate model performance, we employed an independent test set from the German Acute Myeloid Leukemia Cooperative Group (AMLCG) consisting of 40 de novo CN-AML patients aged < 60 years who achieved a complete remission and had RNA-sequencing of their pre-treatment sample. For the training set, there was a significant non-zero cure fraction (*p* = 0.019) with 28.5% of patients estimated to be cured. Our MCM included 112 genes associated with cure, or long-term RFS, and 87 genes associated with latency, or shorter-term time-to-relapse. The area under the curve and C-statistic were respectively, 0.947 and 0.783 for our training set and 0.837 and 0.718 for our test set. We identified a novel, prognostically relevant molecular signature in CN-AML, which allows identification of patient subgroups independent of 2022 ELN genetic-risk groups.

*Trial registration* Data from companion studies CALGB 8461, 9665 and 20202 (trials registered at www.clinicaltrials.gov as, respectively, NCT00048958, NCT00899223, and NCT00900224) were obtained from Alliance for Clinical Trials in Oncology under data sharing study A152010. Data from the AMLCG 2008 trial was registered at www.clinicaltrials.gov as NCT01382147.

**Supplementary Information:**

The online version contains supplementary material available at 10.1186/s13045-024-01553-6.

To the Editor,

Patients with cytogenetically normal AML (CN-AML) comprise the largest cytogenetic subgroup, ranging from 40 to 49% of all adult patients with AML [1]. CN-AML patients are heterogeneous clinically [2] and molecularly [3–8], which has led the European LeukemiaNet (ELN) experts to develop genetic-risk classification, in which the presence of select gene mutations serves as criteria allowing stratification of CN-AML patients into Favorable, Intermediate, and Adverse genetic-risk groups [9]. Kaplan–Meier estimates typically demonstrate a long plateau that does not drop down to zero despite long follow-up, suggesting the existence of a subgroup of CN-AML patients who enjoy long-term relapse-free survival (RFS). In fact, it has been suggested that AML patients attaining 3-year RFS can be considered “potentially cured” [10]. When a Cox proportional hazards model is applied to data that includes a cured subgroup, the hazard and the survival will not be accurately estimated because the proportional hazards assumption is violated [11]. Thus, we used our regularized semi-parametric mixture cure model (MCM) to identify prognostically relevant transcripts that can distinguish CN-AML patients cured from CN-AML patients susceptible with lower- or higher-risk of relapse.

We fit our penalized semi-parametric MCM to our training set, which included 306 adults aged < 60 years (range, 17–59) diagnosed with de novo CN-AML with RNA-sequencing data available and identified 112 genes associated with cure, that is, long-term RFS (Additional file 1: Table S1) and 87 genes associated with latency, that is, shorter-term time-to-relapse (Additional file 1: Table S2). As desired, for the training set the predicted cured group had a survival probability of 1 throughout the observation period, while the predicted susceptible group had an estimated survival curve that descended towards 0 (Fig. 1B). The two risk groups among those predicted to be susceptible were well separated (Fig. 1C). The 5-year area under the curve (AUC) and C-statistic both indicated good predictive ability of our MCM, at 0.947 and 0.783, respectively.

**Fig. 1:**
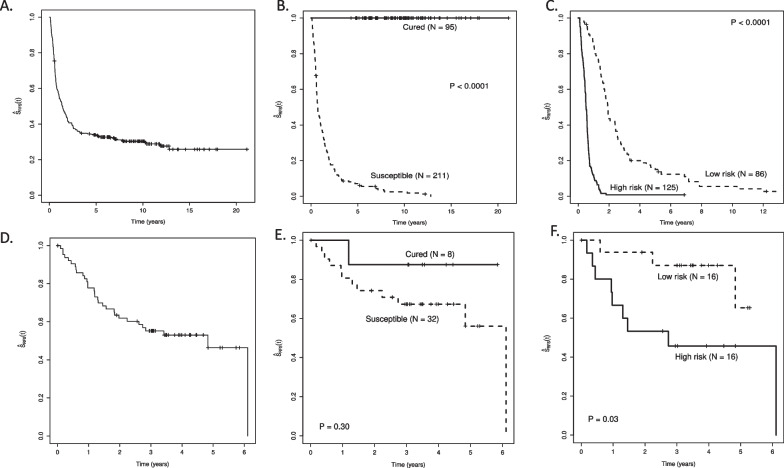
Relapse-free survival for the training set (**A**–**C**) and test set (**D**–**F**). **A** Kaplan–Meier curve for relapse-free survival for the training set. **B** Kaplan–Meier curves for relapse-free survival for the training set stratified by those predicted to be cured versus susceptible to relapse or death using the semi-parametric penalized MCM. **C** Kaplan–Meier curves for relapse-free survival for those predicted to be susceptible in the training set stratified by high versus low risk of relapse using the semi-parametric penalized MCM. **D** Kaplan–Meier curve for relapse-free survival for the test set. **E** Kaplan–Meier curves for relapse-free survival for the test set stratified by those predicted to be cured versus susceptible using the semi-parametric penalized MCM. **F** Kaplan–Meier curves for relapse-free survival for those predicted to be susceptible in the test set stratified by high versus low risk of relapse using the semi-parametric penalized MCM

Only eight of the 40 patients in the independent test set, GSE146173 [12], were predicted to be in the cured group though they had a high survival probability throughout the observation period, with exception of one death at approximately one year (Fig. 1E). Among patients predicted to be susceptible to relapse or death (thereafter referred to as susceptible), there was good separation between the lower- and higher-risk groups (Fig. 1F). The 5-year AUC and C-statistic for the test set both indicated good and relatively good predictive ability of our MCM, at 0.837 and 0.718, respectively.

Interestingly, for our training set, our MCM separated patients predicted to be cured from those predicted to be susceptible in each of the three 2022 ELN genetic-risk groups: Favorable, Intermediate and Adverse (Fig. 2A–C). Despite the small sample sizes for our test set, when performing the same subgroup analyses, there were observable differences in RFS between patients predicted to be cured versus those predicted to be susceptible in all three 2022 ELN genetic-risk groups (Additional file 2: Fig. S1A, B); all patients in the 2022 ELN Adverse genetic-risk group were predicted to be susceptible with higher risk of relapse or death (Additional file 2: Fig. S1C). Moreover, among patients in the training set predicted to be susceptible, RFS differed between those having higher and lower risk in each of the three 2022 ELN genetic-risk groups (Fig. 2D–F). In the test set, RFS also differed between patients predicted to be susceptible having higher versus lower risk for 2022 ELN Favorable, Intermediate, and Adverse genetic-risk groups (Additional file 2: Fig. S2A–C).

**Fig. 2:**
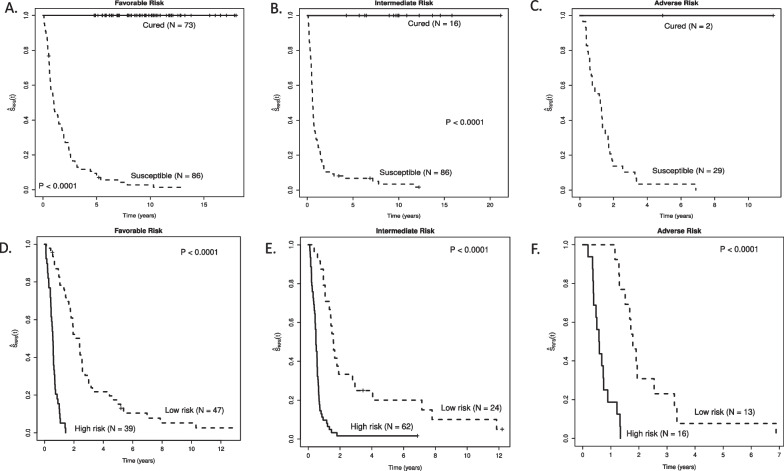
Training set RFS by 2022 ELN, cure status (**A**–**C**), and susceptibility to relapse (**D**–**F**). **A** Kaplan–Meier curves for relapse-free survival for 2022 ELN Favorable risk patients in the training set stratified by those predicted to be cured versus susceptible to relapse or death using the semi-parametric penalized MCM. **B** Kaplan–Meier curves for relapse-free survival for 2022 ELN Intermediate risk patients in the training set stratified by those predicted to be cured versus susceptible using the semi-parametric penalized MCM. **C** Kaplan–Meier curves for relapse-free survival for 2022 ELN Adverse risk patients in the training set stratified by those predicted to be cured versus susceptible using the semi-parametric penalized MCM. **D** Kaplan–Meier curves for relapse-free survival for 2022 ELN Favorable risk patients predicted to be susceptible in the training set using the semi-parametric penalized MCM, stratified by high versus low risk of relapse. **E** Kaplan–Meier curves for relapse-free survival for 2022 ELN Intermediate risk patients predicted to be susceptible in the training set using the semi-parametric penalized MCM, stratified by high versus low risk of relapse. **F** Kaplan–Meier curves for relapse-free survival for 2022 ELN Adverse risk patients predicted to be susceptible in the training set using the semi-parametric penalized MCM, stratified by high versus low risk of relapse

Given that patients in the training set received similar treatment, the identified subgroups (cured, susceptible lower risk, susceptible higher risk) seem to have different sensitivities to 7 + 3-based therapy. Therefore, our work serves as a proof-of-principle that consideration of additional biologic features, such as expression profiles, have the ability to identify patients who have high likelihood of cure with our current standard of care. Thus, our strategy may be useful for refining risk associated with CN-AML patients by identifying those who might be cured with chemotherapy alone and those at higher risk for relapse or death who are in need of different treatment approaches. Future studies should test application of our model a prospective clinical trial and in patients receiving alternative therapies, including those targeting specific gene mutations in CN-AML.

### Supplementary Information

**Additional file 1**: Identifying long-term survivors and those at higher or lower risk of relapse among patients with cytogenetically normal acute myeloid leukemia using a high-dimensional mixture cure model.

**Additional file 2**: Identifying long-term survivors and those at higher or lower risk of relapse among patients with cytogenetically normal acute myeloid leukemia using a high-dimensional mixture cure model.

**Additional file 3**: Identifying long-term survivors and those at higher or lower risk of relapse among patients with cytogenetically normal acute myeloid leukemia using a high-dimensional mixture cure model.

## Data Availability

Data for the training and test sets are summarized in the supplementary information files. The training set is available from the corresponding author on reasonable request. The testing set is available from Gene Expression Omnibus under accession number GSE146173.

## Abbreviations

Alliance: Alliance for Clinical Trials in Oncology
AML: Acute myeloid leukemia
AMLCG: German AML Cooperative Group
AUC: Area under the curve
CALGB: Cancer and Leukemia Group B
CN-AML: Cytogenetically normal acute myeloid leukemia
ELN: European LeukemiaNet
MCM: Mixture cure model
RFS: Relapse-free survival
