# Supplementary material for: Identifying long-term survivors and those at higher or lower risk of relapse among patients with cytogenetically normal acute myeloid leukemia using a high-dimensional mixture cure model
Source: J Hematol Oncol. 2024 May 3;17:28. doi: 10.1186/s13045-024-01553-6 (PMC11068580; doi:10.1186/s13045-024-01553-6)
Supplement: Supplementary file 1 — Additional file 1: Identifying long-term survivors and those at higher or lower risk of relapse among patients with cytogenetically normal acute myeloid leukemia using a high-dimensional mixture cure model. [file 13045_2024_1553_MOESM1_ESM.pdf]

SUPPLEMENTARY TABLES

Supplementary Table 1. Genes included in the incidence portion of the penalized MCM, sorted by the absolute size of their coefficient estimate

ENSEMBL	SYMBOL	MAP	Coefficient estimate
ENSG00000196565	HBG2	11p15.4	-0.430561998
ENSG00000023171	GRAMD1B	11q24.1	0.429745891
ENSG00000225138	SLC9A3-AS1	5p15.33	0.394884275
ENSG00000174130	TLR6	4p14	-0.377969596
ENSG00000197937	ZNF347	19q13.42	-0.370950961
ENSG00000110237	ARHGEF17	11q13.4	0.353799918
ENSG00000113504	SLC12A7	5p15.33	0.349618786
ENSG00000171813	PWWP2B	10q26.3	-0.339856086
ENSG00000180902	D2HGDH	2q37.3	-0.337672361
ENSG00000270562	FOXP1-DT	3p13	0.326274282
ENSG00000185986	SDHAP3	5p15.33	0.310628397
ENSG00000116574	RHOU	1q42.13	0.310061984
ENSG00000119280	C1orf198	1q42.2	-0.306114339
ENSG00000157107	FCHO2	5q13.2	-0.303693144
ENSG00000100299	ARSA	22q13.33	0.293641811
ENSG00000162433	AK4	1p31.3	0.290052543
ENSG00000167291	TBC1D16	17q25.3	0.288430631
ENSG00000139318	DUSP6	12q21.33	0.286192825
ENSG00000187653	TMSB4XP8	4q22.1	-0.276361818
ENSG00000129824	RPS4Y1	Yp11.2	0.266395498
ENSG00000227615	lnc-C5orf49-1	5p15.31	-0.260547237
ENSG00000174080	CTSF	11q13.2	-0.260345979
ENSG00000170909	OSCAR	19q13.42	-0.259063694
ENSG00000186854	TRABD2A	2p11.2	-0.253207202
ENSG00000061918	GUCY1B1	4q32.1	0.251178488
ENSG00000187010	RHD	1p36.11	-0.241462682
ENSG00000115183	TANC1	2q24.2	0.232030292
ENSG00000237892	KLF7-IT1	2q33.3	-0.226363805
ENSG00000188002	PDCD6P1	5p15.33	0.221977176
ENSG00000120594	PLXDC2	10p12.31	-0.221747871
ENSG00000233927	RPS28	19p13.2	0.2182802
ENSG00000104689	TNFRSF10A	8p21.3	-0.215493542
ENSG00000135916	ITM2C	2q37.1	0.214559913
ENSG00000151208	DLG5	10q22.3	-0.207482323
ENSG00000110076	NRXN2	11q13.1	0.204226346
ENSG00000188636	RTL6	22q13.31	-0.202166865
ENSG00000273033	LINC02035	3q21.1	-0.199670391

SUPPLEMENTARY TABLES

ENSEMBL	SYMBOL	MAP	Coefficient estimate
ENSG00000205571	SMN1	5q13.2	-0.172065003
ENSG00000213967	ZNF726	19p12	0.168996404
ENSG00000008283	CYB561	17q23.3	0.166082461
ENSG00000069020	MAST4	5q12.3	0.163413426
ENSG00000114737	CISH	3p21.2	0.156419357
ENSG00000163412	EIF4E3	3p13	0.156175094
ENSG00000188856	RPSAP47	8q21.13	0.151046981
ENSG00000138722	MMRN1	4q22.1	0.150942306
ENSG00000013725	CD6	11q12.2	-0.150604238
ENSG00000169203	NPIPB12	16p11.2	-0.147628971
ENSG00000072422	RHOBTB1	10q21.2	-0.139984836
ENSG00000102409	BEX4	Xq22.1	-0.139172758
ENSG00000171222	SCAND1	20q11.23	-0.137726519
ENSG00000133401	PDZD2	5p13.3	0.137402276
ENSG00000124882	EREG	4q13.3	0.133316677
ENSG00000187627	RGPD1	2p11.2	0.129229778
ENSG00000150782	IL18	11q23.1	0.122336099
ENSG00000115525	ST3GAL5	2p11.2	0.119416483
ENSG00000226471	lnc-CCDC117-1	22q12.1	0.118535255
ENSG00000106526	ACTR3C	7q36.1	-0.118278227
ENSG00000156381	ANKRD9	14q32.31	-0.117696098
ENSG00000100596	SPTLC2	14q24.3	-0.113051469
ENSG00000168939	SPRY3	Xq28	-0.112379482
ENSG00000250251	PKD1P6	16p13.11	-0.112093048
ENSG00000197561	ELANE	19p13.3	0.11133613
ENSG00000090975	PITPNM2	12q24.31	-0.110561164
ENSG00000058056	USP13	3q26.33	-0.109812651
ENSG00000123080	CDKN2C	1p32.3	-0.103617481
ENSG00000260727	SLC7A5P1	16p11.2	-0.102057577
ENSG00000100916	BRMS1L	14q13.2	-0.09951764
ENSG00000111275	ALDH2	12q24.12	0.096757729
ENSG00000238160	LINC02863	5q31.1	0.094861526
ENSG00000237298	TTN-AS1	2q31.2	-0.093030226
ENSG00000153563	CD8A	2p11.2	-0.09250286
ENSG00000080823	MOK	14q32.31	0.091887433
ENSG00000001561	ENPP4	6p21.1	-0.087311244
ENSG00000144647	POMGNT2	3p22.1	-0.085952923
ENSG00000152767	FARP1	13q32.2	0.085703253
ENSG00000244405	ETV5	3q27.2	-0.084042931
ENSG00000121274	TENT4B	16q12.1	-0.083872651

SUPPLEMENTARY TABLES

ENSEMBL	SYMBOL	MAP	Coefficient estimate
ENSG00000175265	GOLGA8A	15q14	0.082845902
ENSG00000236876	TMSB4XP1	1p34.2	-0.08135629
ENSG00000271646	IRF2-DT	4q35.1	0.080288763
ENSG00000247627	MTND4P12	5q31.1	-0.079562269
ENSG00000170456	DENND5B	12p11.21	-0.077641624
ENSG00000100376	FAM118A	22q13.31	0.076055753
ENSG00000214425	LRRC37A4P	17q21.31	-0.076040705
ENSG00000007062	PROM1	4p15.32	0.069657787
ENSG00000049192	ADAMTS6	5q12.3	-0.063941645
ENSG00000259276	HSALNT0382748	15q25.3	0.057791547
ENSG00000106780	MEGF9	9q33.2	-0.047079761
ENSG00000145476	CYP4V2	q35.2	-0.046914218
ENSG00000060138	YBX3	12p13.2	0.04614826
ENSG00000170035	UBE2E3	2q31.3	-0.046083115
ENSG00000132819	RBM38	20q13.31	-0.046009468
ENSG00000132122	SPATA6	1p33	-0.042365658
ENSG00000234883	MIR155HG	21q21.3	0.040561418
ENSG00000107957	SH3PXD2A	10q24.33	-0.038625494
ENSG00000130702	LAMA5	20q13.33	-0.033321365
ENSG00000233974	POM121 pseudogene	5p14.3	-0.032612698
ENSG00000264895	HSALNG0145324	17q21.33	-0.027950985
ENSG00000182871	COL18A1	21q22.3	-0.027394183
ENSG00000068489	PRR11	17q22	-0.026359848
ENSG00000110799	VWF	12p13.31	0.026154387
ENSG00000147180	ZNF711	Xq21.1	0.026050318
ENSG00000166435	XRRA1	11q13.4	-0.024337407
ENSG00000223345	H2BP1	1p11.2	-0.020735752
ENSG00000157240	FZD1	7q21.13	-0.014418459
ENSG00000144677	CTDSPL	3p22.2	0.011354679
ENSG00000167851	CD300A	17q25.1	0.009623768
ENSG00000189223	PAX8-AS1	2q14.1	0.00676482
ENSG00000120675	DNAJC15	13q14.11	-0.005296614
ENSG00000174059	CD34	1q32.2	0.003554299
ENSG00000229153	EPHA1-AS1	7q35	-0.000636884
ENSG00000134531	EMP1	12p13.1	0.000237136

SUPPLEMENTARY TABLES

Supplementary Table 2. Genes included in the latency portion of the penalized MCM, sorted by the absolute size of their coefficient estimate

ENSEMBL	SYMBOL	MAP	Coefficient estimate
ENSG00000174059	CD34	1q32.2	0.38890555
ENSG00000173083	HPSE	4q21.23	-0.34084587
ENSG00000273018	FAM106A	17p11.2	-0.305979777
ENSG00000254415	SIGLEC14	19q13.41	-0.292543578
ENSG00000071894	CPSF1	8q24.3	-0.278074177
ENSG00000197253	TPSB2	16p13.3	-0.277988443
ENSG00000168502	MTCL1	18p11.22	0.261079608
ENSG00000076555	ACACB	12q24.11	-0.228209685
ENSG00000179630	LACC1	13q14.11	-0.225571164
ENSG00000181104	F2R	5q13.3	0.221809537
ENSG00000117480	FAAH	1p33	0.218580954
ENSG00000173530	TNFRSF10D	8p21.3	0.210230749
ENSG00000131188	PRR7	5q35.3	0.209063364
ENSG00000140287	HDC	15q21.2	-0.207754375
ENSG00000149131	SERPING1	11q12.1	-0.192533729
ENSG00000132849	PATJ	1p31.3	-0.1881949
ENSG00000136997	MYC	8q24.21	0.188085348
ENSG00000196415	PRTN3	19p13.3	0.178134316
ENSG00000135362	PRR5L	11p13-p12	0.174424911
ENSG00000029639	TFB1M	6q25.3	0.174393482
ENSG00000241489	B3KWA1	Xq28	0.173229675
ENSG00000213722	DDAH2	6p21.33	-0.173080816
ENSG00000124882	EREG	4q13.3	0.166567918
ENSG00000127152	BCL11B	14q32.2	-0.158897988
ENSG00000100448	CTSG	14q12	0.157138941
ENSG00000159788	RGS12	4p16.3	0.156858263
ENSG00000136193	SCRN1	7p14.3	0.15170139
ENSG00000229153	EPHA1-AS1	7q35	-0.144263562
ENSG00000159403	C1R	12p13.31	0.143339188
ENSG00000175352	NRIP3	11p15.4	-0.142716001
ENSG00000213906	LTB4R2	14q12	0.141642333
ENSG00000008405	CRY1	12q23.3	-0.131412539
ENSG00000018189	RUFY3	4q13.3	-0.130426824
ENSG00000167851	CD300A	17q25.1	0.129639937
ENSG00000124019	FAM124B	2q36.2	0.128077973
ENSG00000185986	SDHAP3	5p15.33	0.126654694
ENSG00000170180	GYPA	4q31.21	-0.124036206

SUPPLEMENTARY TABLES

ENSEMBL	SYMBOL	MAP	Coefficient estimate
ENSG00000204172	AGAP9	10q11.22	-0.121690607
ENSG00000208772	SNORD94	2p11.2	0.121682699
ENSG00000166987	MBD6	12q13.3	0.120165881
ENSG00000197081	IGF2R	6q25.3	0.11852073
ENSG00000166435	XRRA1	11q13.4	-0.117591022
ENSG00000227191	TRGC2	7p14.1	0.113761037
ENSG00000131370	SH3BP5	3p25.1	0.113356178
ENSG00000188636	RTL6	22q13.31	-0.111961905
ENSG00000132530	XAF1	17p13.1	0.106931602
ENSG00000106546	AHR	7p21.1	0.106767262
ENSG00000200243	RN7SKP79	5p15.31	-0.098834878
ENSG00000148444	COMMD3	10p12.2	0.095382434
ENSG00000180596	H2BC4	6p22.2	-0.094772903
ENSG00000070404	FSTL3	19p13.3	0.094291261
ENSG00000205593	DENND6B	22q13.33	0.090209897
ENSG00000152778	IFIT5	10q23.31	0.089023219
ENSG00000185129	PURA	5q31.3	-0.088919446
ENSG00000199347	RNU5E-1	1p36.22	0.078872961
ENSG00000163814	CDCP1	3p21.31	0.077981334
ENSG00000230076	RPL10P6	2q35	-0.075186255
ENSG00000231721	LINC-PINT	7q32.3	0.071324547
ENSG00000144036	EXOC6B	2p13.2	-0.070114731
ENSG00000137193	PIM1	6p21.2	0.067305593
ENSG00000023909	GCLM	1p22.1	-0.062437159
ENSG00000072422	RHOBTB1	10q21.2	-0.05986443
ENSG00000201801	RNU5E-4P	1p36.22	0.056169274
ENSG00000185883	ATP6V0C	16p13.3	0.049542986
ENSG00000120913	PDLIM2	8p21.3	0.047626731
ENSG00000196565	HBG2	11p15.4	-0.044737899
ENSG00000089723	OTUB2	14q32.12	0.043260914
ENSG00000214016	RPSAP61	Xp11.3	-0.04225761
ENSG00000151208	DLG5	10q22.3	-0.040464746
ENSG00000261377	PDCD6IPP2	15q13.1	0.035766321
ENSG00000185710	SMG1P4	16p12.2	-0.031606669
ENSG00000105808	RASA4	7q22.1	-0.031388851
ENSG00000253276	CCDC71L	7q22.3	0.02941447
ENSG00000154217	PITPNC1	17q24.2	-0.023085786
ENSG00000213626	LBH	2p23.1	-0.021526643
ENSG00000022267	FHL1	Xq26.3	0.020973797
ENSG00000196155	PLEKHG4	16q22.1	-0.017251314

SUPPLEMENTARY TABLES

ENSEMBL	SYMBOL	MAP	Coefficient estimate
ENSG00000223345	H2BP1	1p11.2	-0.015800561
ENSG00000121039	RDH10	8q21.11	0.0152843
ENSG00000188211	NCR3LG1	11p15.1	-0.01372034
ENSG00000213085	CFAP45	1q23.2	-0.01171965
ENSG00000135272	MDFIC	q31.2	0.005847174
ENSG00000253210	DENND3-AS1	8q24.3	0.005781547
ENSG00000241529	RB7SL767P	3q13.2	-0.0042305
ENSG00000112773	TENT5A	6q14.1	0.002917112
ENSG00000100418	DESI1	22q13.2	-0.002221063
ENSG00000102409	BEX4	Xq22.1	-0.001633697

SUPPLEMENTARY TABLES

Supplementary Table 3. Genes included in both the incidence and latency portions of the penalized MCM, sorted by the maximum absolute size of their coefficient estimates

ENSEMBL	SYMBOL	MAP	Incidence coefficient estimate	Latency coefficient estimate	Maximum absolute coefficient estimate
ENSG00000196565	HBG2	11p15.4	-0.430562	-0.0447379	0.430562
ENSG00000174059	CD34	1q32.2	0.0035543	0.38890555	0.38890555
ENSG00000185986	SDHAP3	5p15.33	0.3106284	0.12665469	0.3106284
ENSG00000151208	DLG5	10q22.3	-0.2074823	-0.0404647	0.20748232
ENSG00000188636	RTL6	22q13.31	-0.2021669	-0.1119619	0.20216687
ENSG00000124882	EREG	4q13.3	0.13331668	0.16656792	0.16656792
ENSG00000229153	AS1	7q35	-0.0006369	-0.1442636	0.14426356
ENSG00000072422	RHOBTB1	10q21.2	-0.1399848	-0.0598644	0.13998484
ENSG00000102409	BEX4	Xq22.1	-0.1391728	-0.0016337	0.13917276
ENSG00000167851	CD300A	17q25.1	0.00962377	0.12963994	0.12963994
ENSG00000166435	XRRA1	11q13.4	-0.0243374	-0.117591	0.11759102
ENSG00000223345	H2BP1	1p11.2	-0.0207358	-0.0158006	0.02073575

SUPPLEMENTARY TABLES

Supplementary Table 4. Demographic, clinical, and gene mutation data in the training (Alliance) and test (AMLCG) sets

**Characteristic***	**Training set n=306**	**Test set n=40**	***p*-value**
Age, years	46.0 (17 - 59)	45.5 (18 - 57)	0.598
Sex			
Female	147 (48.0)	27 (67.5)	0.028
Male	159 (52.0)	13 (32.5)	
Hemoglobin, g/dL	9.2 (4.8 – 25.1)	9.15 (5.1 – 14.0)	0.447
Platelet count, x10^9/L	57 (8 - 445)	61 (10 - 265)	0.225
White blood cell count, x10^9/L	28.6 (0.6 - 475)	17.6 (1.6 - 156)	0.199
Bone marrow blasts, %	68.5 (10 - 96)	77.5 (26 - 95)	0.021
Blood blasts, %	60.0 (0 - 97)	24.0 (0 - 95)	<0.001
2022 ELN genetic group, n (%)			0.749
Favorable	159 (54.5)	19 (48.7)	
Intermediate	102 (34.9)	16 (41.0)	
Adverse	31 (10.6)	4 (10.3)	
DNMT3A			1.00
Mutated	123 (41.3)	16 (40.0)	
Wild-type	175 (58.7)	24 (60.0)	
NRAS			0.025
Mutated	45 (15.1)	12 (30.0)	
Wild-type	253 (84.9)	28 (70.0)	
SF3B1			1.00
Mutated	7 (2.4)	0 (0.0)	
Wild-type	290 (97.6)	40 (100.0)	
IDH1			0.399
Mutated	31 (10.4)	2 (5.0)	
Wild-type	267 (89.6)	38 (95.0)	
CEBPA^{bZIP}			0.266
Mutated	55 (18.5)	4 (10.0)	
Wild-type	243 (81.5)	36 (90.0)	
GATA2			0.774
Mutated	27 (9.1)	4 (10.0)	
Wild-type	271 (90.9)	36 (90.0)	
TET2			0.278
Mutated	29 (9.7)	6 (15.0)	
Wild-type	269 (90.3)	34 (85.0)	
NPM1			1.00

SUPPLEMENTARY TABLES

Characteristic*	Training set n=306	Test set n=40	*p*-value
Mutated	192 (64.4)	26 (65.0)	
Wild-type	106 (35.6)	14 (35.0)	
WT1			<0.001
Mutated	19 (6.4)	12 (30.0)	
Wild-type	279 (93.6)	28 (70.0)	
PTPN11			1.00
Mutated	31 (10.4)	4 (10.0)	
Wild-type	267 (89.6)	36 (90.0)	
FLT3-TKD			0.069
Present	33 (11.1)	9 (22.5)	
Absent	264 (88.9)	31 (77.5)	
FLT3-ITD			0.724
Present	103 (34.1)	15 (37.5)	
Absent	199 (65.9)	25 (62.5)	
IDH2			1.00
Mutated	31 (10.4)	4 (10.0)	
Wild-type	267 (89.6)	36 (90.0)	
MLL-PTD			0.272
Present	6 (5.2)	4 (10.3)	
Absent	110 (94.8)	35 (89.7)	
TP53			1.00
Mutated	1 (0.3)	0 (0.0)	
Wild-type	297 (99.7)	40 (100.0)	
SRSF2			1.00
Mutated	10 (3.4)	1 (2.5)	
Wild-type	286 (96.6)	39 (97.5)	
ASXL1			0.378
Mutated	13 (4.4)	0 (0.0)	
Wild-type	285 (95.6)	40 (100.0)	
RUNX1			0.437
Mutated	14 (4.7)	3 (7.5)	
Wild-type	284 (95.3)	37 (92.5)	
BCOR			0.336
Mutated	8 (2.7)	2 (5.0)	
Wild-type	290 (97.3)	38 (95.0)	

ELN, European LeukemiaNet.

* Continuous variables were summarized by reporting the median (range) while categorical variables were summarized by reporting frequency (percentage).

SUPPLEMENTARY TABLES

Supplementary Table 5. Demographic, clinical, and gene mutation data in the training set categorized by the penalized mixture cure model as cured or susceptible with low risk or high risk

Characteristic*	Cured n=95	Low risk n=86	High risk n=125	*p*-value
Age, years	45 (18 - 59)	44 (19 - 59)	47 (17 - 59)	0.681
Sex				
Female	45 (47.4)	40 (46.5)	62 (49.6)	0.906
Male	50 (52.6)	46 (53.5)	63 (50.4)	
Hemoglobin, g/dL	9.4 (4.9 – 25.1)	9.35 (4.9 – 13.6)	9.1 (4.8 – 13.7)	0.207
Platelet count, x10^9/L	55.0 (8 - 445)	64.5 (9 - 433)	56.0 (13 - 266)	0.262
White blood cell count, x10^9/L	24.9 (1.9 - 475)	22.5 (0.6 – 295.0)	34.4 (1.0 – 308.8)	0.02
Bone marrow blasts, %	64 (10 - 93)	68.5 (20 - 95)	70 (18 - 96)	0.018
Blood blasts, %	55 (0 - 95)	60.5 (0 - 97)	65 (0 - 97)	0.087
2022 ELN genetic group, n (%)				<0.001
Favorable	73 (80.2)	47 (56.0)	39 (33.3)	
Intermediate	16 (17.6)	24 (28.6)	62 (53.0)	
Adverse	2 (2.2)	13 (15.5)	16 (13.7)	
DNMT3A				<0.001
Mutated	33 (35.1)	24 (28.2)	66 (55.5)	
Wild-type	61 (64.9)	61 (71.8)	53 (44.5)	
NRAS				0.446
Mutated	18 (19.1)	11 (12.9)	16 (13.4)	
Wild-type	76 (80.9)	74 (87.1)	103 (86.6)	
SF3B1				0.119
Mutated	0 (0.0)	2 (2.4)	5 (4.2)	
Wild-type	94 (100.0)	82 (97.6)	114 (95.8)	
IDH1				0.503
Mutated	7 (7.4)	9 (10.6)	15 (12.6)	
Wild-type	87 (92.6)	76 (89.4)	104 (87.4)	
CEBPA^{bZIP}				0.035
Mutated	25 (26.6)	15 (17.6)	15 (12.6)	
Wild-type	69 (73.4)	70 (82.4)	104 (87.4)	
GATA2				0.169
Mutated	13 (13.8)	6 (7.1)	8 (6.7)	
Wild-type	81 (86.2)	79 (92.9)	111 (93.3)	
TET2				0.532
Mutated	10 (10.6)	10 (11.8)	9 (7.6)	
Wild-type	84 (89.4)	75 (88.2)	110 (92.4)	

SUPPLEMENTARY TABLES

**Characteristic***	**Cured n=95**	**Low risk n=86**	**High risk n=125**	***p*-value**
NPM1				0.372
Mutated	66 (70.2)	53 (62.4)	73 (61.3)	
Wild-type	28 (29.8)	32 (37.6)	46 (38.7)	
WT1				0.096
Mutated	2 (2.1)	7 (8.2)	10 (8.4)	
Wild-type	92 (97.9)	78 (91.8)	109 (91.6)	
PTPN11				0.073
Mutated	14 (14.9)	10 (11.8)	7 (5.9)	
Wild-type	80 (85.1)	75 (88.2)	112 (94.1)	
FLT3-TKD				0.005
Present	18 (19.4)	9 (10.6)	6 (5.0)	
Absent	75 (80.6)	76 (89.4)	113 (95.0)	
FLT3-ITD				<0.001
Present	15 (15.8)	22 (26.2)	66 (53.7)	
Absent	80 (84.2)	62 (73.8)	57 (46.3)	
IDH2				0.121
Mutated	7 (7.4)	14 (16.5)	10 (8.4)	
Wild-type	87 (92.6)	71 (83.5)	109 (91.6)	
MLL-PTD				0.772
Present	3 (7.7)	1 (2.9)	2 (4.8)	
Absent	36 (92.3)	34 (97.1)	40 (95.2)	
TP53				0.601
Mutated	1 (1.1)	0 (0.0)	0 (0.0)	
Wild-type	93 (98.9)	85 (100.0)	119 (100.0)	
SRSF2				0.282
Mutated	1 (1.1)	4 (4.8)	5 (4.2)	
Wild-type	92 (98.9)	80 (95.2)	114 (95.8)	
ASXL1				0.937
Mutated	4 (4.3)	3 (3.5)	6 (5.0)	
Wild-type	90 (95.7)	82 (96.5)	113 (95.0)	
RUNX1				0.036
Mutated	1 (1.1)	3 (3.5)	10 (8.4)	
Wild-type	93 (98.9)	82 (96.5)	109 (91.6)	
BCOR				0.353
Mutated	1 (1.1)	2 (2.4)	5 (4.2)	
Wild-type	93 (98.9)	83 (97.6)	114 (95.8)	
Number of mutations	2 (0, 5)	2.5 (0, 4)	3.5 (0, 5)	0.196

ELN, European LeukemiaNet.

SUPPLEMENTARY TABLES

* Continuous variables were summarized by reporting median (range) while categorical variables were summarized by reporting frequency (percentage).

SUPPLEMENTARY TABLES

Supplementary Table 6. Demographic, clinical, and gene mutation data in the test set categorized by the penalized mixture cure model as cured or susceptible with low risk or high risk

Characteristic*	Cured n=8	Low risk n=16	High risk n=16	*p*-value
Age, years	43.5 (18 - 54)	45.5 (25 - 56)	47.5 (19 - 57)	0.818
Sex				0.516
Female	4 (50.0)	11 (68.8)	12 (75.0)	
Male	4 (50.0)	5 (31.2)	4 (25.0)	
Hemoglobin, g/dL	9.8 (5.1 – 13.8)	9.25 (5.1 – 11.2)	8.4 (6.8 – 14.0)	0.716
Platelet count, x10^9/L	47.5 (13 - 265)	63 (10 – 128)	55 (18 - 238)	0.816
White blood cell count, x10^9/L	13.9 (6.4 – 50.9)	12.9 (1.6 – 146.4)	20.9 (2.96 - 156)	0.436
Bone marrow blasts, %	90 (35 - 95)	86 (30 - 93)	74 (26 -95)	0.835
Blood blasts, %	1 (0 - 94)	25 (0 - 95)	34 (0 - 80)	0.232
2022 ELN genetic group, n (%)				0.637
Favorable	6 (75.0)	7 (43.8)	6 (40.0)	
Intermediate	2 (25.0)	7 (43.8)	7 (46.7)	
Adverse	0 (0.0)	2 (12.5)	2 (13.3)	
DNMT3A				0.496
Mutated	2 (25.0)	6 (37.5)	8 (50.0)	
Wild-type	6 (75.0)	10 (62.5)	8 (50.0)	
NRAS				0.155
Mutated	4 (50.0)	2 (12.5)	6 (37.5)	
Wild-type	4 (50.0)	14 (87.5)	10 (62.5)	
SF3B1				NA
Mutated	0 (0.0)	0 (0.0)	0 (0.0)	
Wild-type	8 (100.0)	16 (100.0)	16 (100.0)	
IDH1				0.672
Mutated	1 (12.5)	1 (6.2)	0 (0.0)	
Wild-type	7 (87.5)	15 (93.8)	16 (100.0)	
CEBPA^{bZIP}				0.020
Mutated	3 (37.5)	0 (0.0)	1 (6.2)	
Wild-type	5 (62.5)	16 (100.0)	15 (93.8)	
GATA2				0.02
Mutated	3 (37.5)	0 (0.0)	1 (6.2)	
Wild-type	5 (62.5)	16 (100.0)	15 (93.8)	
TET2				0.167
Mutated	3 (37.5)	1 (6.2)	2 (12.5)	
Wild-type	5 (62.5)	15 (93.8)	14 (87.5)	
NPM1				0.576

SUPPLEMENTARY TABLES

Characteristic*	Cured n=8	Low risk n=16	High risk n=16	*p*-value
Mutated	5 (62.5)	12 (75.0)	9 (56.2)	
Wild-type	3 (37.5)	4 (25.0)	7 (43.8)	
WT1				0.017
Mutated	1 (12.5)	2 (12.5)	9 (56.2)	
Wild-type	7 (87.5)	14 (87.5)	7 (43.8)	
PTPN11				1.00
Mutated	1 (12.5)	2 (12.5)	1 (6.2)	
Wild-type	7 (87.5)	14 (87.5)	15 (93.8)	
FLT3 TKD				0.791
Present	1 (12.5)	4 (25.0)	4 (25.0)	
Absent	7 (87.5)	12 (75.0)	12 (75.0)	
FLT3 ITD				0.522
Present	2 (25.0)	5 (31.2)	8 (50.0)	
Absent	6 (75.0)	11 (68.8)	8 (50.0)	
IDH2				0.506
Mutated	0 (0.0)	3 (18.8)	1 (6.2)	
Wild-type	8 (100.0)	13 (81.2)	15 (93.8)	
MLL-PTD				0.406
Present	0 (0.0)	1 (6.2)	3 (20.0)	
Absent	8 (100.0)	15 (93.8)	12 (80.0)	
TP53				NA
Mutated	0 (0.0)	0 (0.0)	0 (0.0)	
Wild-type	8 (100.0)	16 (100.0)	16 (100.0)	
SRSF2				1.00
Mutated	0 (0.0)	1 (6.2)	0 (0.0)	
Wild-type	8 (100.0)	15 (93.8)	16 (100.0)	
ASXL1				NA
Mutated	0 (0.0)	0 (0.0)	0 (0.0)	
Wild-type	8 (100.0)	16 (100.0)	16 (100.0)	
RUNX1				0.793
Mutated	0 (0.0)	1 (6.2)	2 (12.5)	
Wild-type	8 (100.0)	15 (93.8)	14 (87.5)	
BCOR				0.344
Mutated	0 (0.0)	0 (0.0)	2 (12.5)	
Wild-type	8 (100.0)	16 (100.0)	14 (87.5)	
Number of mutations	3 (1, 5)	3 (0, 4)	3.5 (1, 7)	0.199

* Continuous variables were summarized by reporting median (range) while categorical variables were summarized by reporting frequency (percentage).
