# Supplementary material for: Identifying long-term survivors and those at higher or lower risk of relapse among patients with cytogenetically normal acute myeloid leukemia using a high-dimensional mixture cure model
Source: J Hematol Oncol. 2024 May 3;17:28. doi: 10.1186/s13045-024-01553-6 (PMC11068580; doi:10.1186/s13045-024-01553-6)

Supplementary Figure 1. RFS by 2022 ELN genetic-risk group and cure status for the test set. A) Kaplan-Meier curve for relapse-free survival for 2022 ELN Favorable risk patients in the test set stratified by those predicted to be cured versus susceptible using the semi-parametric penalized MCM. B) Kaplan-Meier curve for relapse-free survival for 2022 ELN Intermediate risk patients in the test set stratified by those predicted to be cured versus susceptible using the semi-parametric penalized MCM. C) Kaplan-Meier curve for relapse-free survival for 2022 ELN Adverse risk patients in the test set stratified by those predicted to be cured versus susceptible using the semi-parametric penalized MCM. **.**

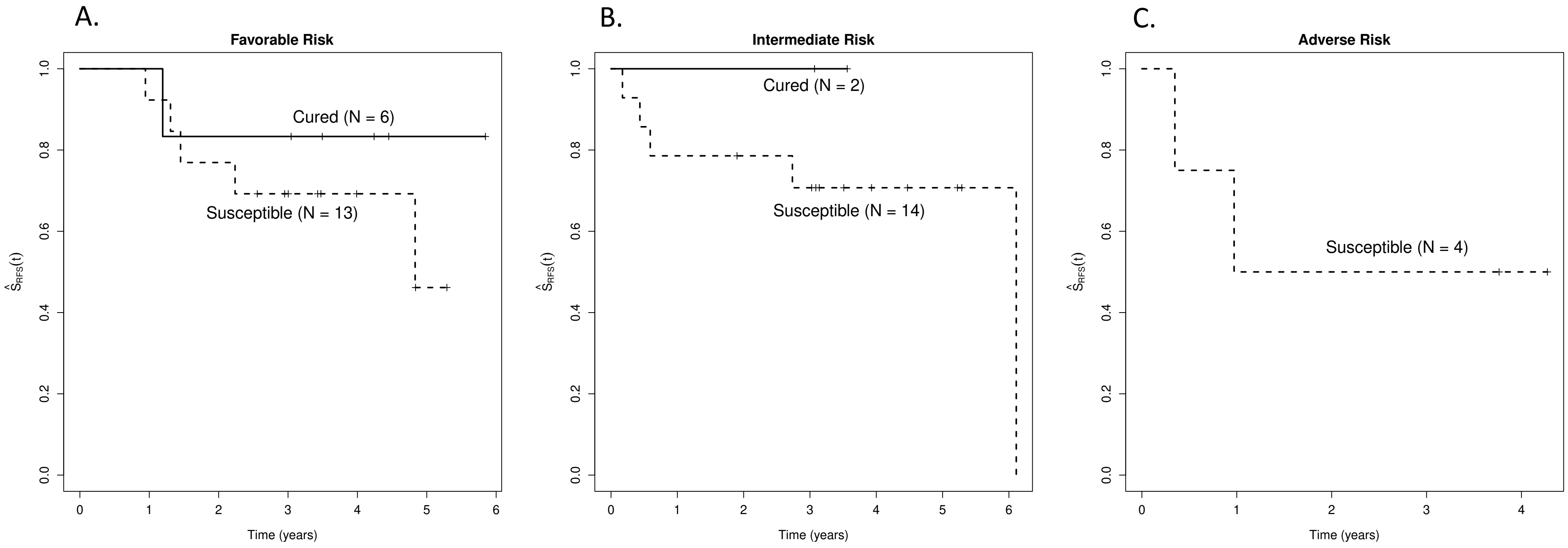

Supplementary Figure 2. RFS by 2022 ELN genetic-risk group among susceptibles in the test set. A) Kaplan-Meier curve for relapse-free survival for 2022 ELN Favorable risk patients predicted to be susceptible in the test set using the semi-parametric penalized MCM, stratified by high versus low risk of relapse. B) Kaplan-Meier curve for relapse-free survival for 2022 ELN Intermediate risk patients predicted to be susceptible in the test set using the semi-parametric penalized MCM, stratified by high versus low risk of relapse. C) Kaplan-Meier curve for relapse-free survival for 2022 ELN Adverse risk patients predicted to be susceptible in the test set using the semi-parametric penalized MCM, stratified by high versus low risk of relapse.

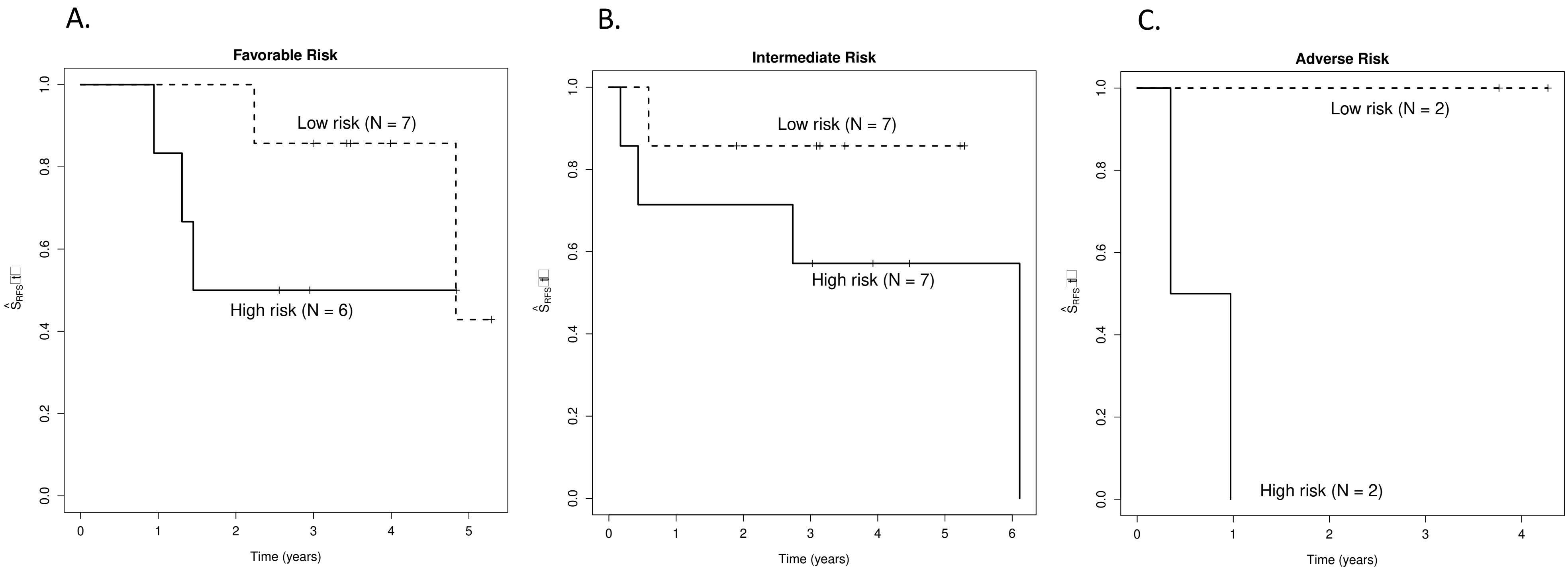

Supplementary Figure 3. A) Kaplan-Meier curve for relapse-free survival for the training set stratified by those predicted to be cured versus susceptible using the semi-parametric penalized MCM among those with *NPM1* mutant. B) Kaplan-Meier curve for relapse-free survival for those predicted to be susceptible in the training set stratified by high versus low risk of relapse using the semi-parametric penalized MCM among those with *NPM1* mutant.

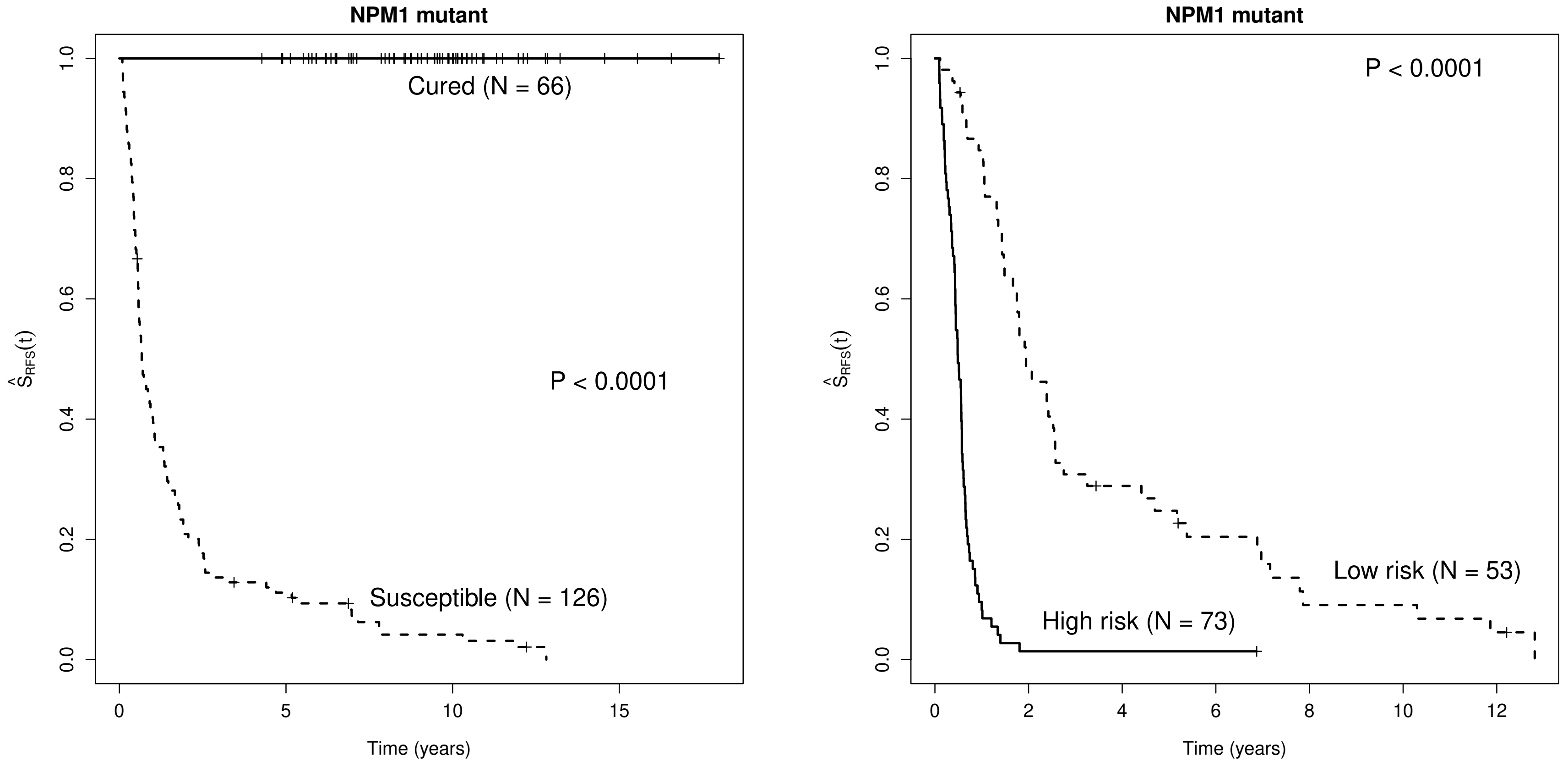

Supplementary Figure 4. A) Kaplan-Meier curve for relapse-free survival for the training set stratified by those predicted to be cured versus susceptible using the semi-parametric penalized MCM among those with *CEBPA*^{bzip}. B) Kaplan-Meier curve for relapse-free survival for those predicted to be susceptible in the training set stratified by high versus low risk of relapse using the semi-parametric penalized MCM among those with *CEBPA*^{bzip}.

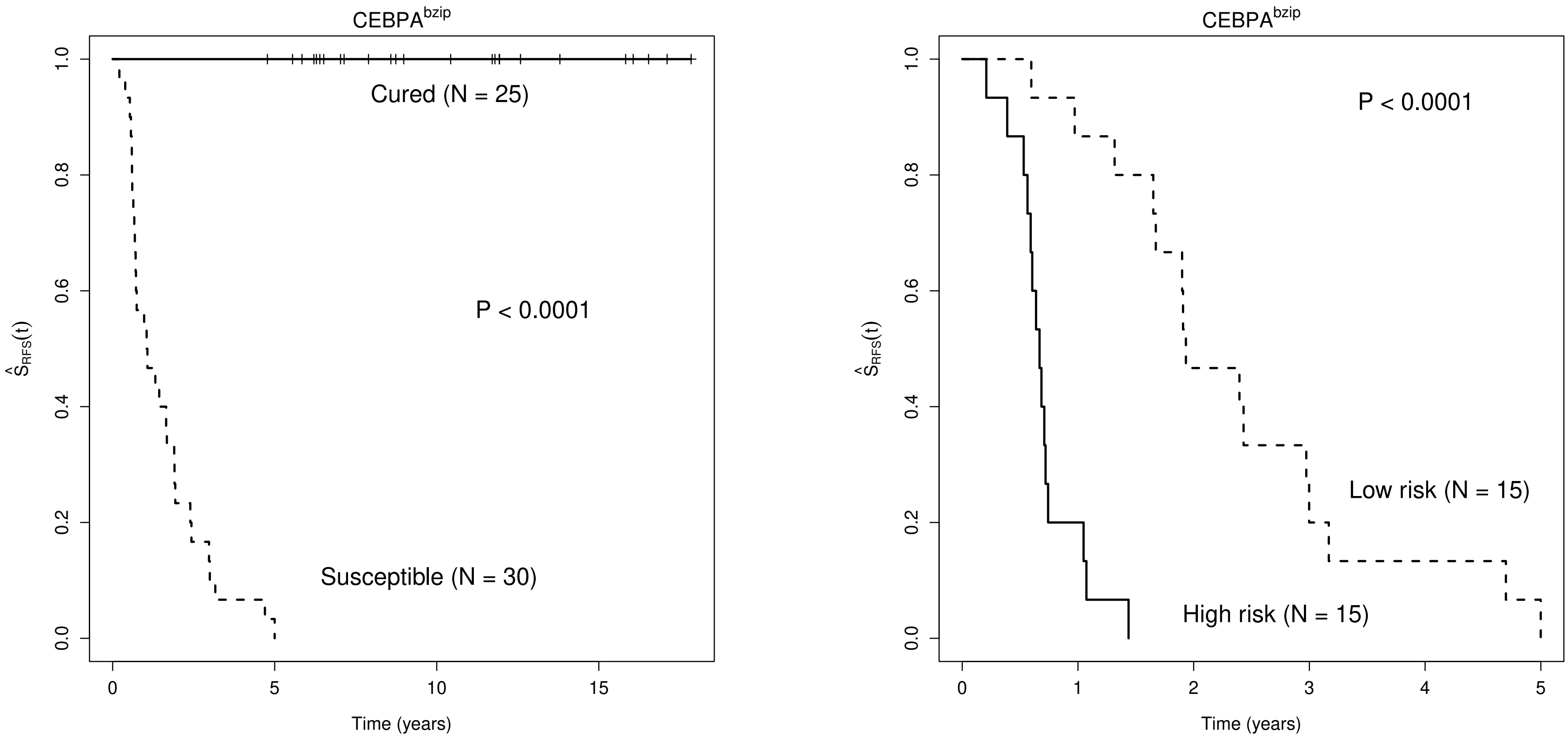

Supplementary Figure 5. A) Kaplan-Meier curve for relapse-free survival for the training set stratified by those predicted to be cured versus susceptible using the semi-parametric penalized MCM among those with *DNMT3A* mutant. B) Kaplan-Meier curve for relapse-free survival for those predicted to be susceptible in the training set stratified by high versus low risk of relapse using the semi-parametric penalized MCM among those with *DNMT3A* mutant.

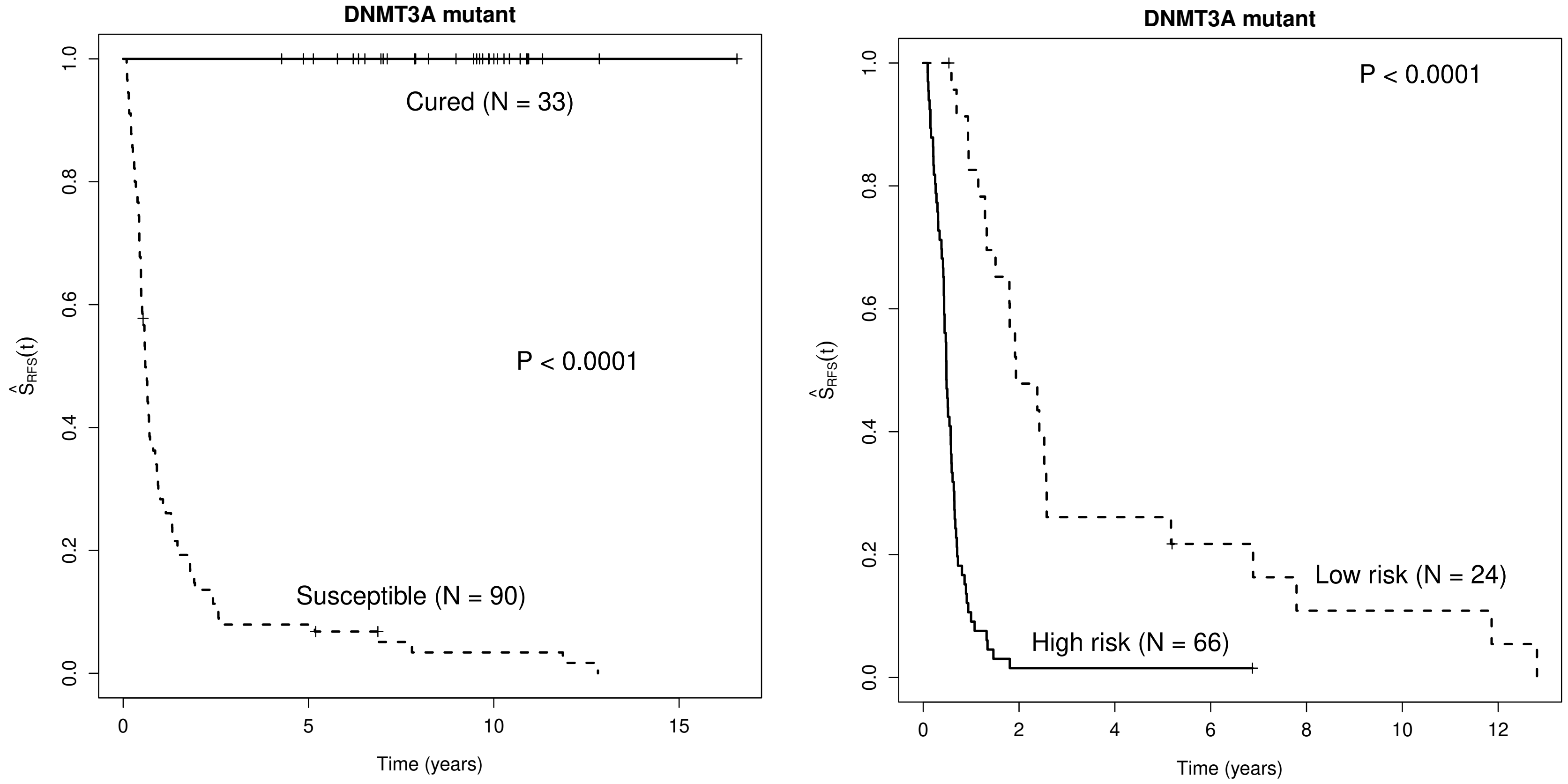

Supplementary Figure 6. A) Kaplan-Meier curve for relapse-free survival for the training set stratified by those predicted to be cured versus susceptible using the semi-parametric penalized MCM among those with *FLT3*-ITD. B) Kaplan-Meier curve for relapse-free survival for those predicted to be susceptible in the training set stratified by high versus low risk of relapse using the semi-parametric penalized MCM among those with *FLT3*-ITD.

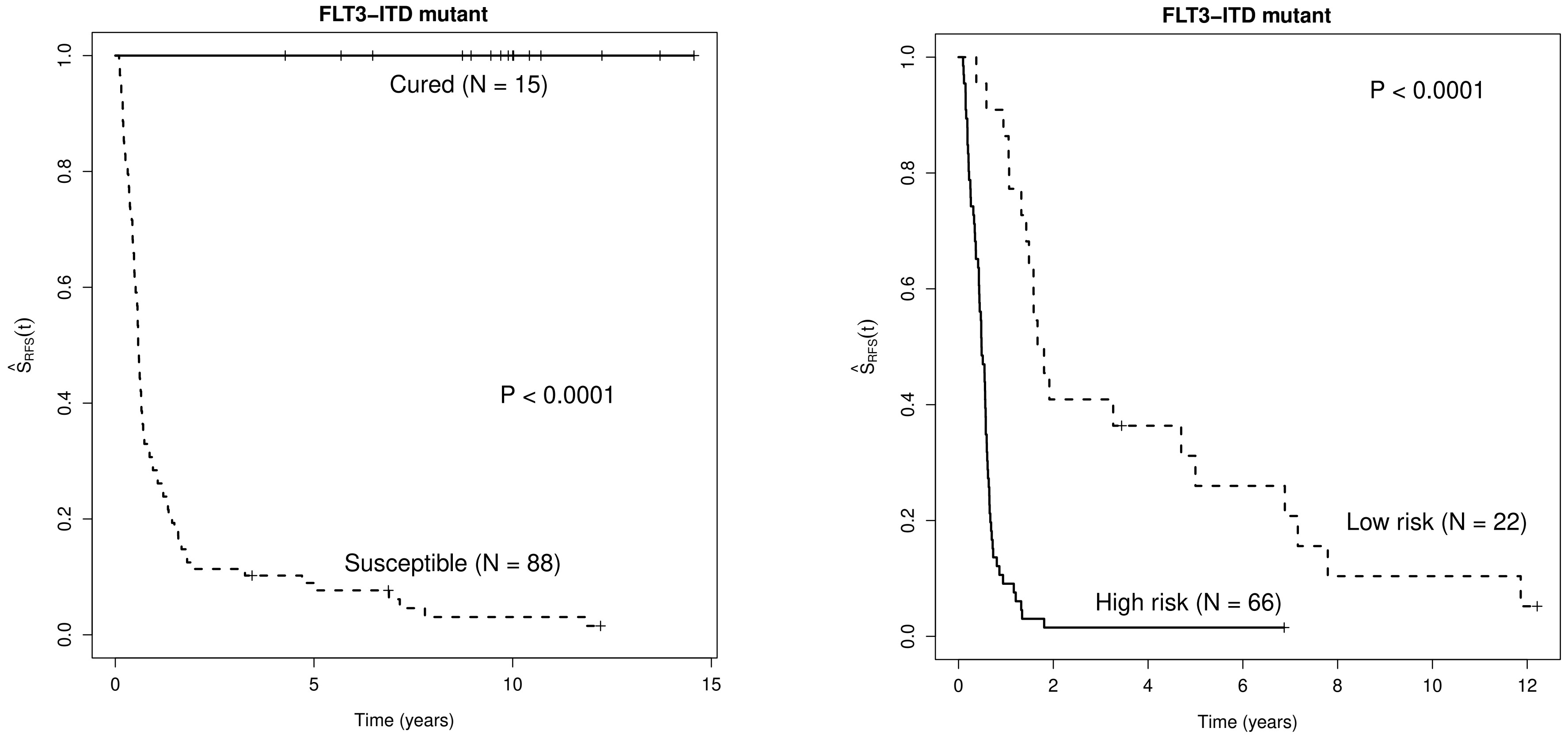

Supplementary Figure 7. A) Kaplan-Meier curve for relapse-free survival for the training set stratified by those predicted to be cured versus susceptible using the semi-parametric penalized MCM among those with *FLT3*-TKD. B) Kaplan-Meier curve for relapse-free survival for those predicted to be susceptible in the training set stratified by high versus low risk of relapse using the semi-parametric penalized MCM among those with *FLT3*-TKD.

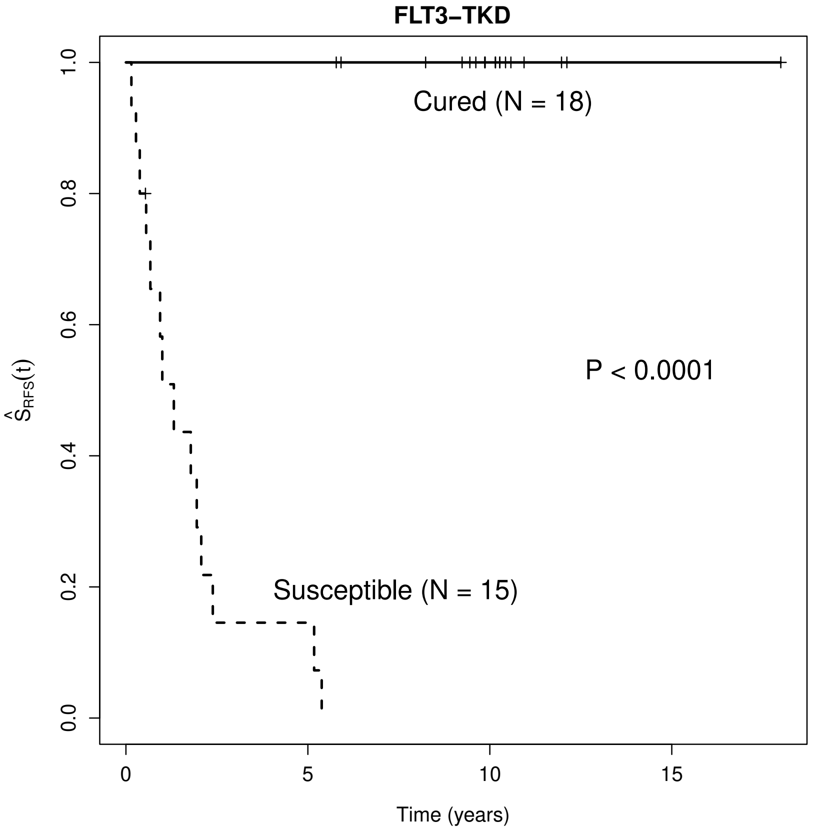

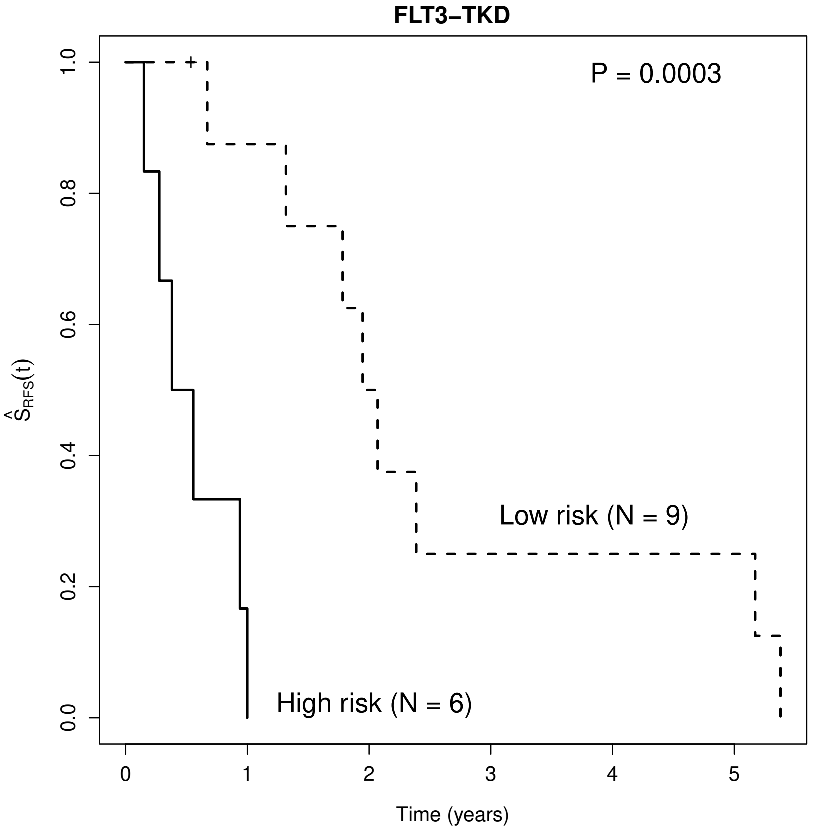

Supplementary Figure 8. A) Kaplan-Meier curve for relapse-free survival for the training set stratified by those predicted to be cured versus susceptible using the semi-parametric penalized MCM among those with *IDH1* mutant. B) Kaplan-Meier curve for relapse-free survival for those predicted to be susceptible in the training set stratified by high versus low risk of relapse using the semi-parametric penalized MCM among those with *IDH1* mutant.

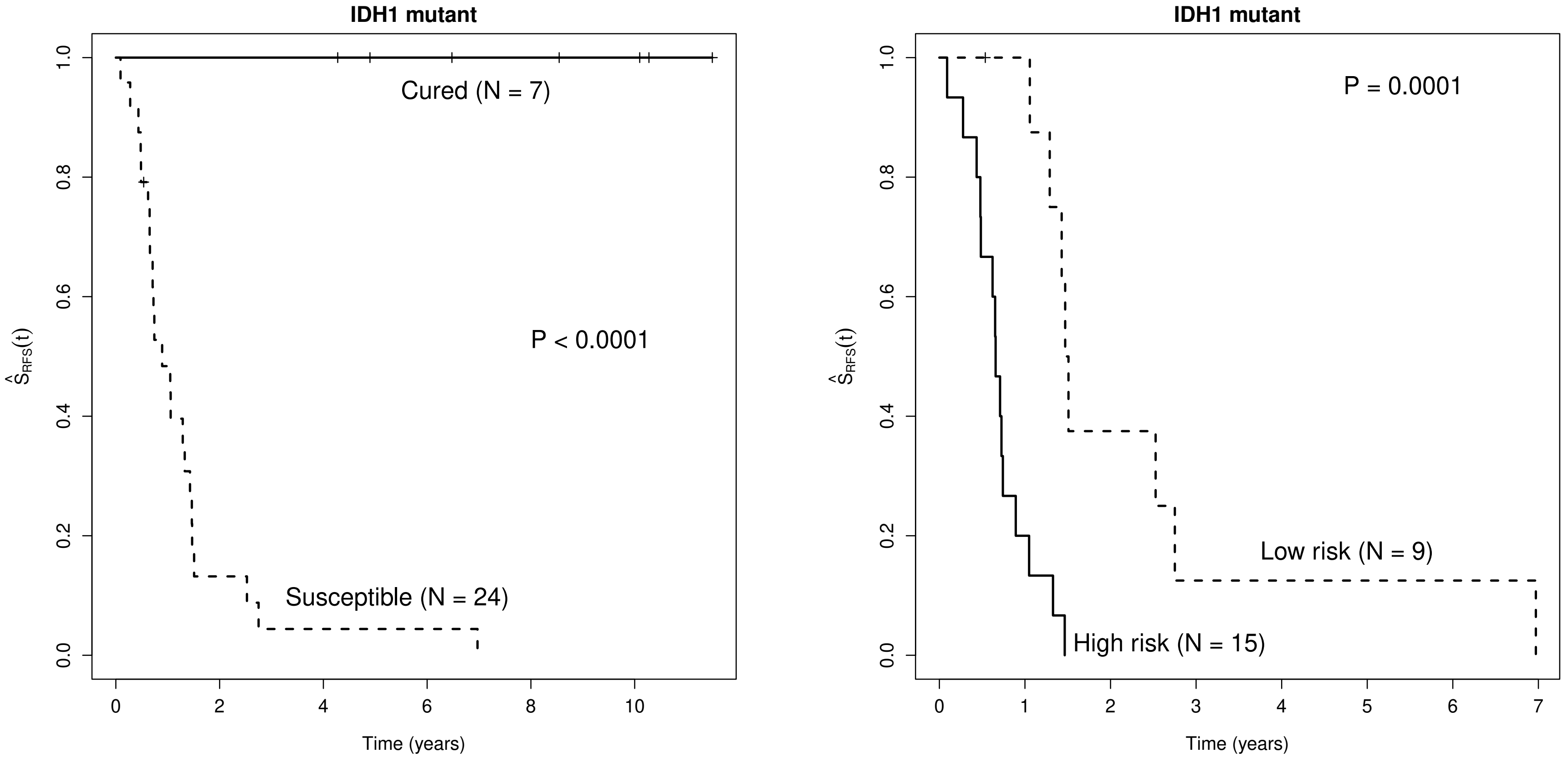

Supplementary Figure 9. A) Kaplan-Meier curve for relapse-free survival for the training set stratified by those predicted to be cured versus susceptible using the semi-parametric penalized MCM among those with *IDH2* mutant. B) Kaplan-Meier curve for relapse-free survival for those predicted to be susceptible in the training set stratified by high versus low risk of relapse using the semi-parametric penalized MCM among those with *IDH2* mutant.

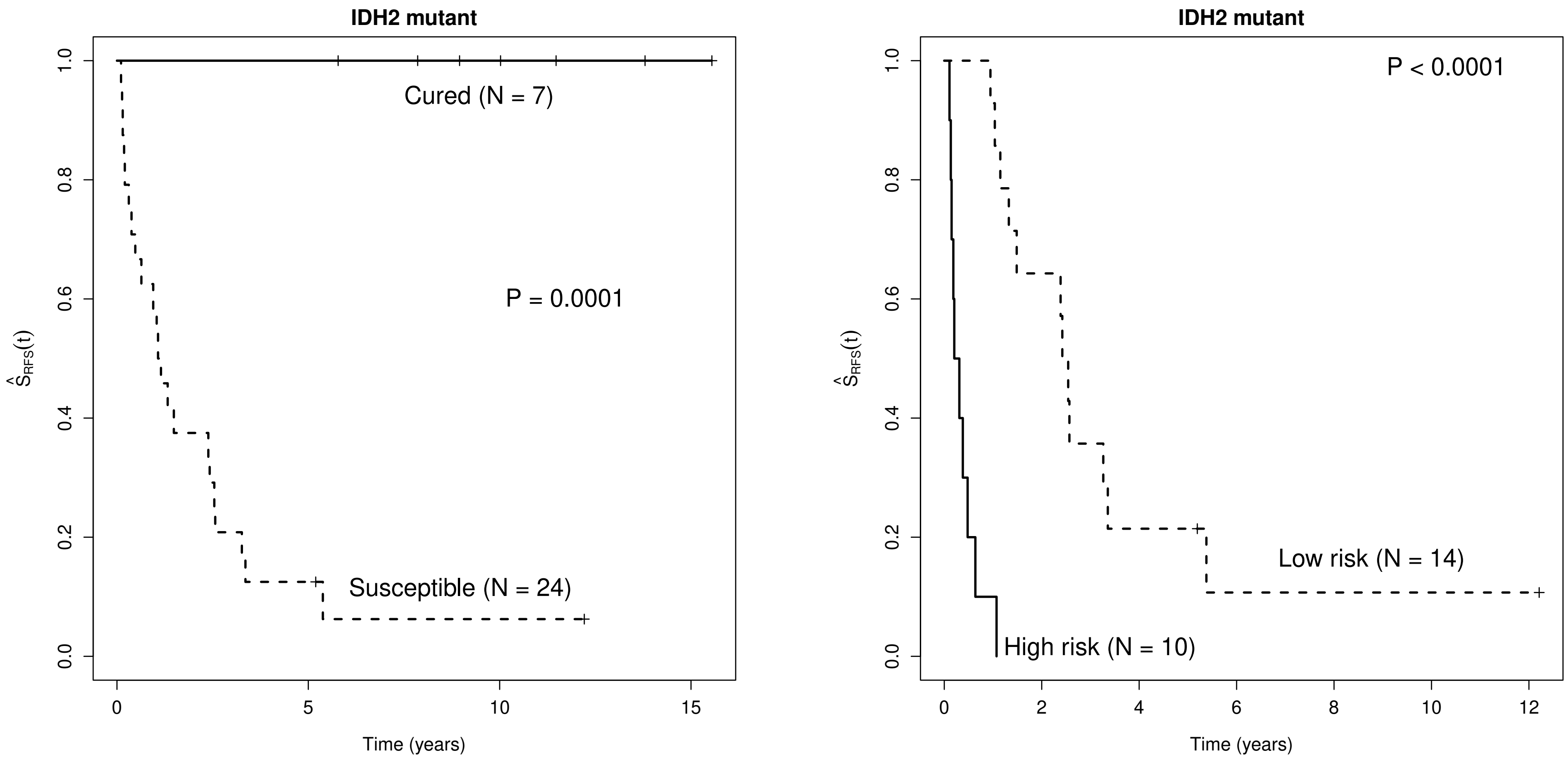

Supplementary Figure 10. A) Kaplan-Meier curve for relapse-free survival for the training set stratified by those predicted to be cured versus susceptible using the semi-parametric penalized MCM among those with *NRAS* mutant. B) Kaplan-Meier curve for relapse-free survival for those predicted to be susceptible in the training set stratified by high versus low risk of relapse using the semi-parametric penalized MCM among those with *NRAS* mutant.

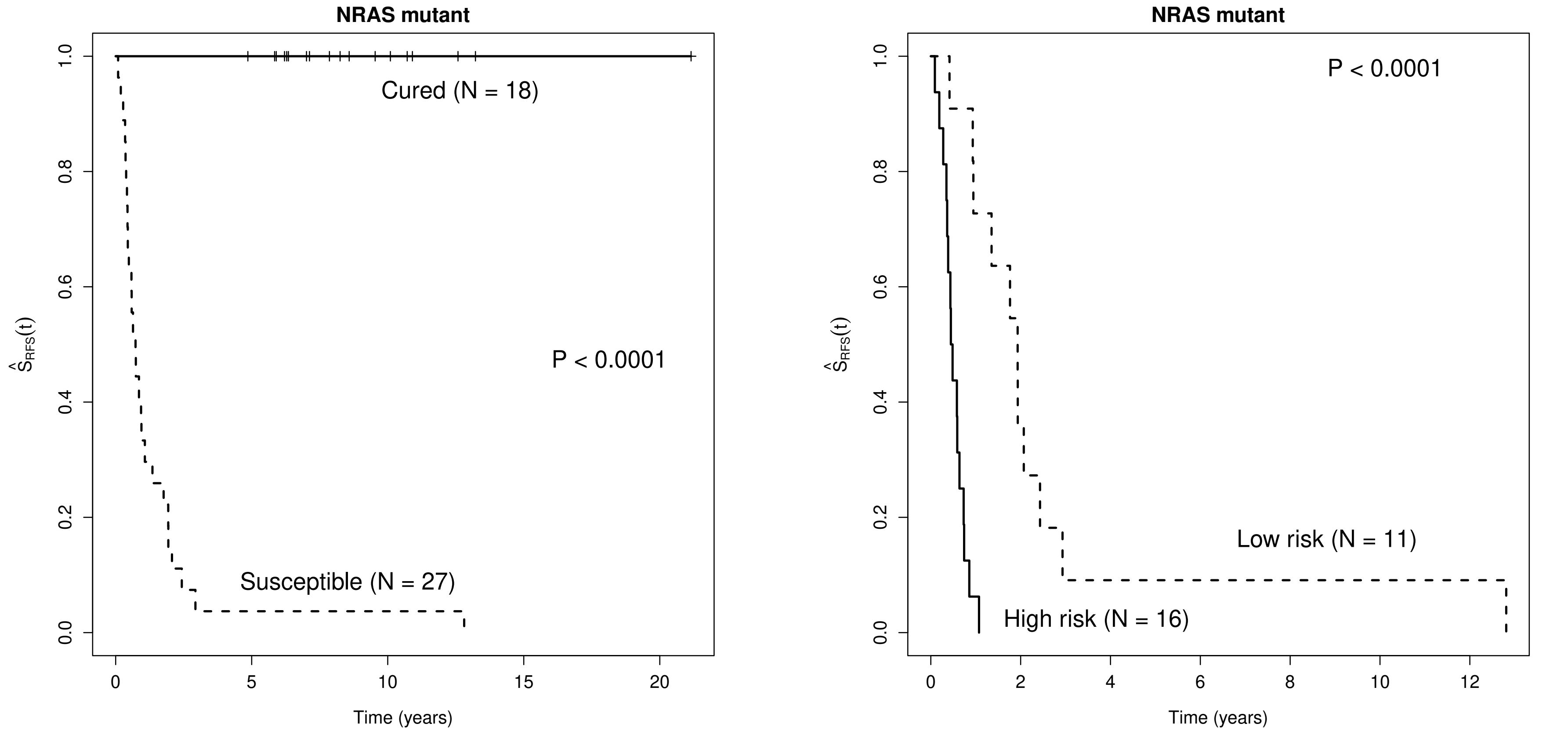

Supplementary Figure 11. A) Kaplan-Meier curve for relapse-free survival for the training set stratified by those predicted to be cured versus susceptible using the semi-parametric penalized MCM among those with *PTPN11* mutant. B) Kaplan-Meier curve for relapse-free survival for those predicted to be susceptible in the training set stratified by high versus low risk of relapse using the semi-parametric penalized MCM among those with biallelic *PTPN11* mutant.

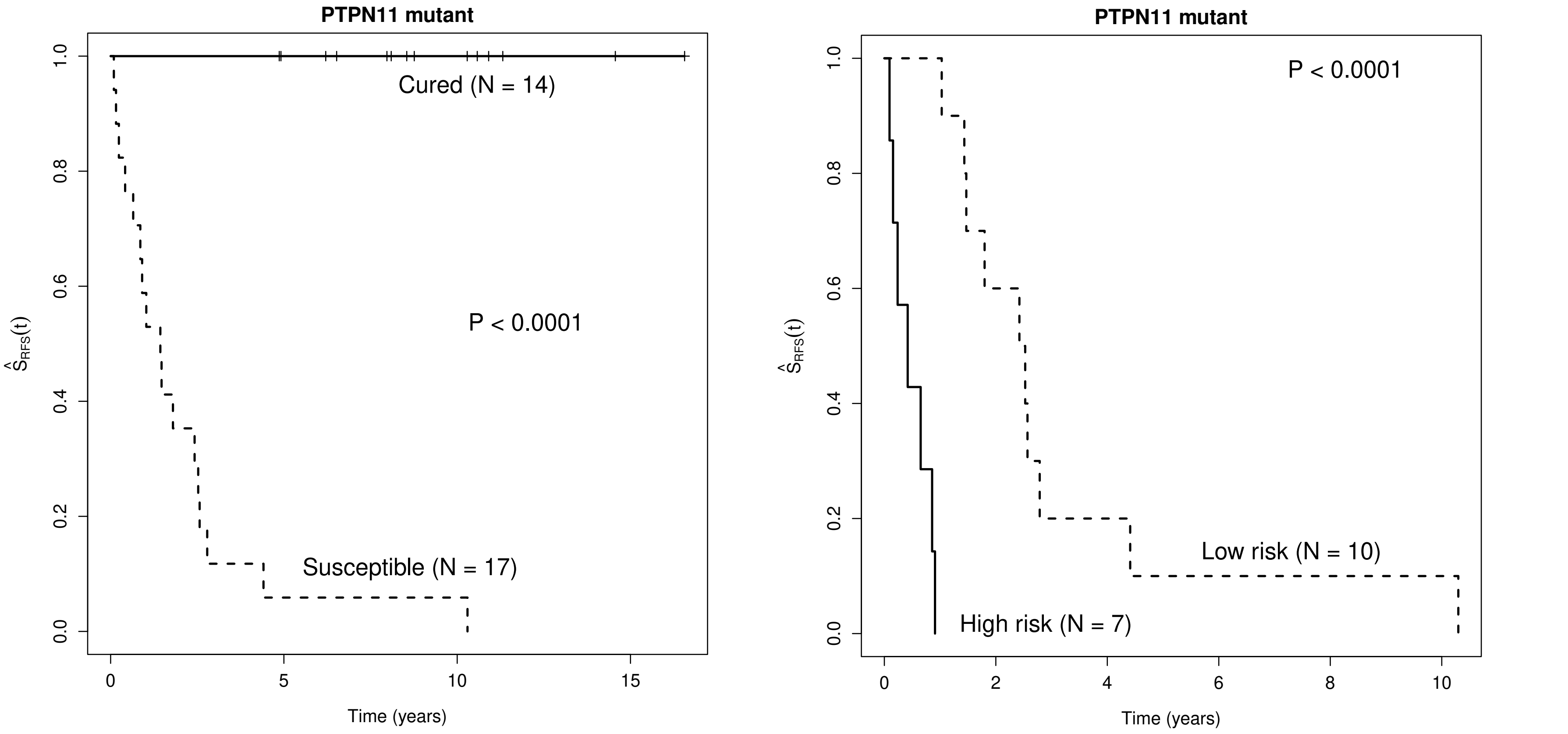

Supplement: Supplementary file 2 — Additional file 2: Identifying long-term survivors and those at higher or lower risk of relapse among patients with cytogenetically normal acute myeloid leukemia using a high-dimensional mixture cure model. [file 13045_2024_1553_MOESM2_ESM.pdf]
