# Supplementary material for: Identifying long-term survivors and those at higher or lower risk of relapse among patients with cytogenetically normal acute myeloid leukemia using a high-dimensional mixture cure model
Source: J Hematol Oncol. 2024 May 3;17:28. doi: 10.1186/s13045-024-01553-6 (PMC11068580; doi:10.1186/s13045-024-01553-6)
Supplement: Supplementary file 3 — Additional file 3: Identifying long-term survivors and those at higher or lower risk of relapse among patients with cytogenetically normal acute myeloid leukemia using a high-dimensional mixture cure model. [file 13045_2024_1553_MOESM3_ESM.pdf]

Identifying long-term survivors and those at higher or lower risk of relapse among patients with cytogenetically normal acute myeloid leukemia using a high-dimensional mixture cure model

Kellie J. Archer[1]*, Han Fu[2], Krzysztof Mrózek[3], Deedra Nicolet[3,4], Alice S. Mims[3], Geoffrey L. Uy[5], Wendy Stock[6], John C. Byrd[7], Wolfgang Hiddemann[8], Jan Braess[9], Karsten Spiekermann[8], Klaus H. Metzeler[10], Tobias Herold[8] and Ann-Kathrin Eisfeld[3]

[1] Division of Biostatistics, College of Public Health, The Ohio State University, Columbus, OH, USA

[2] Google, Inc., Mountain View, CA, USA

[3] Clara D. Bloomfield Center for Leukemia Outcomes Research, The Ohio State University Comprehensive Cancer Center, Columbus, OH, USA

[4] Alliance Statistics and Data Management Center, The Ohio State University Comprehensive Cancer Center, Columbus, OH, USA

[5] Department of Medicine, Division of Oncology, Washington University School of Medicine, St. Louis, MO, USA

[6] Department of Medicine, Section of Hematology/Oncology, University of Chicago, Chicago, IL, USA

[7] Department of Internal Medicine, University of Cincinnati, Cincinnati, OH, USA

[8] Laboratory for Leukemia Diagnostics, Department of Medicine III, University Hospital, LMU Munich, Munich, Germany

[9] Department of Oncology and Hematology, Hospital Barmherzige Brüder, Regensburg, Germany

[10] Department of Hematology and Cellular Therapy, University Hospital Leipzig, Leipzig, Germany

Methods

Patients and treatment

Prognosis in AML is associated with age [1,2] and the treatment used, therefore we analyzed younger CN-AML patients aged <60 years, for whom cytotoxic induction chemotherapy is standard treatment. Our training set included 306 adult patients aged <60 years (range, 17-59) diagnosed with *de novo* AML between 1986 and 2016 and enrolled on frontline Cancer and Leukemia Group B (CALGB) clinical trials and companion studies. CALGB is now part of Alliance for Clinical Trials in Oncology (Alliance). All patients were cytogenetically normal, which was determined in each case by analysis of ≥20 metaphase cells in bone marrow (BM) specimens subjected to short-term (24- and/or 48-hour) culture performed by CALGB-approved institutional laboratories. The results were confirmed by central karyotype review [3]. Generally, patients received intensive cytarabine and daunorubicin or idarubicin-based induction treatment and all patients included achieved a complete remission (CR). No patient received allogeneic hematopoietic stem-cell transplantation (HSCT) in first CR on study protocols, and patients who were transplanted off-study were excluded because of missing or incomplete follow-up data. All patients had RNA-sequencing and gene mutation data from diagnostic samples available. Gene mutation profiling was performed based on a custom-designed 80 gene panel, that includes the recurrently mutated, repeatedly validated AML-associated genes based on published disease-defining hallmark studies [4,5]. To validate the performance of our fitted model, we employed an independent test set from the German AML Cooperative Group (AMLCG), available under Gene Expression Omnibus accession number GSE146173 [6], where we restricted attention to the 40 *de*

novo CN-AML patients aged <60 years who were treated in the AMLCG 2008 trial [7], achieved a CR (excluding patients who achieved CR with incomplete blood recovery), and had gene expression measured in their pre-treatment samples using the RNA-seq. Mutation data for the AMLCG cohort were available for 19 genes – to enforce consistency between the training and test sets, we restricted attention to these 19 gene mutations. All patients provided written informed consent for participation in the treatment studies. Institutional Review Board approval of all CALGB/Alliance and AMLCG protocols was obtained before any research was performed.

RNA-Seq preprocessing methods

Training set RNA-seq sample preparation

The training set of *de novo* CN-AML patients aged <60 years who achieved complete remission was derived from a larger dataset that initially consisted of 893 samples from patients with abnormal AML or CN-AML, 29 CBF patients from the Nationwide cohort, and 49 patients from the Nationwide Disparities AML Project. Tumor derived RNA was subjected to 0.8x SPRI bead cleanup and size selection prior to DNase treatment and ribodepletion prior to using the NEBNext Ultra II Directional kit preparation. The library was constructed for whole transcriptome sequencing (RNA-seq). Paired-end reads were either 50-bp or 150-bp and were generated on the Illumina NovaSeq (Illumina, Inc.). Prior to alignment, 150-bp reads were trimmed to 50-bp to mitigate batch effects due to read length. Then reads were aligned to the human genome reference sequence (GRCh38).

Training set RNA-seq data pre-processing

Samples with < 10 million total counts and/or < 60% of total counts assigned to protein coding genes were removed from the dataset. Two batches were defined: as 893 samples from the Alliance for Clinical Trials in Oncology (Alliance; consisting of abnormal AML and CN-AML patients) and 78 samples from two Nationwide Projects (CBF and Disparities AML Projects). Genes having a mean counts-per-million ≥ 0.5 in at least one batch were retained. The R package poibm (installed from https://bitbucket.org/anthakki/poibm/src/master/ on March 2, 2023) was used for batch correction. The poibm.fit function was called with the count data in the Nationwide batch (source) and Alliance batch (target space). The phenotype parameters were set to rhoX = 0.25 and rhoY=1; parameter values controlling the E-M estimation were set to max.iter = 100 and max.resets = 20. The poibm.apply function was used to apply the fits (corrections) to the Nationwide sample counts, followed by rounding to the nearest integer.

Clinical endpoints and statistical analysis

We estimated RFS as the time from CR until relapse, death, or last-follow-up, censoring for patients alive without relapse. Following the 2022 ELN guidelines [8], we considered both relapse and death as events when estimating RFS. Therefore, herein “cure” is synonymous with attaining long-term RFS. Prior to applying an MCM, we needed to verify we had sufficient follow-up [9,10] and that there was a significant non-zero cure fraction in our dataset. To this end, we examined the Kaplan-Meier survival curve to ascertain the plateau and estimated the time at which 95% of events should

occur [11]. We then performed a hypothesis test to detect whether there was a significant non-zero cure fraction for RFS using both the simulated non-parametric and the parametric approaches [11,12].

We were interested in investigating the effects of the covariates on our time-to-event outcome. We hypothesized that there was one subgroup consisting of patients immune to the event (those relapse-free, in other words, those who were cured) and another subgroup consisting of patients susceptible (termed susceptibles) to the event (relapse or death). We let π represent the proportion of susceptibles, so (1 - π) represents the proportion cured. The finite mixture model can be used to arrive at the overall RFS model, $S(t)$, which is decomposed into the sum of the individual subgroup survival functions weighted by the proportion of the relevant subgroup size, or

$$S(t) = (1 - \pi)S_c(t) + \pi S_s(t). \qquad \text{(Eq. 1)}$$

where $S_c(t)$ is the survival function for those cured while $S_s(t)$ is the survival function for those susceptible [12], We also note that for those cured, the survival function, $S_c(t)$, at any timepoint is 1, which can be substituted into Eq. 1 to yield a simplified survival function. As previously mentioned, we were interested in estimating the effect of covariates on the outcome. Notice again that our outcome model (Eq. 1) consists of two components: the incidence component which models susceptible versus cured and the latency component which models time-to-event for those susceptible. It is possible that different covariates affect these different parts of our model, so we let **x** and **w** denote the covariates included in the incidence and latency components, respectively. Thus, our MCM

$$S(t|\mathbf{x}, \mathbf{w}) = \big(1 - \pi(\mathbf{x})\big) + \pi(\mathbf{x})S_s(t, \mathbf{w}|Y = 1) \qquad \text{(Eq. 2)}$$

allows us to identify the effect of covariates on the likelihood of being susceptible to the event versus immune to the event (or cured) and the time-to-event (or latency) among those susceptible. The latency or time-to-event function for susceptibles can be modeled in different ways, using, for example, a parametric, a non-parametric, or semi-parametric survival model. We choose to use the semi-parametric Cox model as it relaxes the parametric assumptions in the baseline latency distribution and therefore increases model flexibility over parametric MCMs [13].

Our goal was to identify a multivariable model using genomic features, namely genes having expression significantly associated with either the cure or latency. Because few variable selection methods exist for high-dimensional MCMs, we developed a penalized semi-parametric MCM for high-dimensional datasets to identify prognostically relevant genes that can distinguish patients cured from patients susceptible to the event with lower or higher risk of relapse. Given our observed data and unknown parameters, we used the Expectation-Maximization (E-M) algorithm [14] where we divided the problem into logistic regression for cure status component and survival regression for susceptible patients to estimate the parameters for the latency component of the model. Specifically, we expressed the complete-data log-likelihood assuming the cure status is observed and included a LASSO penalty for the incidence covariates and a LASSO penalty for the penalized latency covariates. Initial values for the intercept for the incidence portion of the model and the baseline hazard for the latency portion of the model were obtained by fitting logistic regression and survival models, respectively, and all penalized coefficients were initialized to 0. In the Estimation step (E-step), we calculated the expected penalized complete-data log-

likelihood with respect to the conditional distribution of cure given the current parameter estimates and observed data, where we replaced the unknown cure status indicator with its estimated proportion. In the Maximization step (M-step), we estimated the parameters by expressing the objective function as the sum of two separate functions corresponding to the incidence and latency components of the model. The incidence component was estimated as a penalized logistic regression with a fractional response. The latency component involves the parameters and the baseline latency function, so the optimization problem turned into a penalized Cox regression with an offset term. The baseline latency was estimated by first obtaining Breslow's estimator for the cumulative baseline hazard though inclusion of an extra term in the denominator and applying the Weibull tail completion method when the last observation is censored. The E-M algorithm alternated between an E-step and a M-step until convergence.

Mixture cure model applied to the training and test sets

RNA-seq expression values were candidate covariates in our semi-parametric penalized MCM to identify a parsimonious list of transcripts associated with cure or latency. Pre-processing steps applied to the RNA-seq data for the training set are described in the Training Set RNA-seq Data Pre-processing section. The AMLCG RNA-seq data were processed as previously described [6]. We combined the training and test sets then voom normalized [15] and applied ComBat [16] to batch-correct RNA-Seq data, then filtered the data by applying thresholds to gene-level means (>2) and standard deviations (>0.75) of our training set, leaving 4,770 genes for model fitting. Ten-fold cross validation was repeated 100 times to choose the optimal model using the

training set, using the cross-validated area under the receiver operating characteristic curve (AUC) to select the penalty for the incidence portion of the model and the cross-validated C-statistic to select the penalty for the latency portion of the model. After fitting our penalized semi-parametric MCM to the training set, we applied the fitted MCM to the test set.

Results

Demographic, clinical, and select gene mutation data for patients in our training and test sets are presented in Supplementary Table 4. The training set was more balanced with respect to sex than the test set (p=0.028), and the test set patients had significantly higher percentage of BM blasts (p=0.021), whereas the training set patients had significantly higher percentage of blasts in the blood (p<0.001). Among 19 gene mutations examined, there were differences in the frequencies of *NRAS* (p=0.025) and *WT1* (p<0.001) mutations between the training and test sets, whereas there was no significant difference with respect to the patient assignment into the 2022 ELN genetic-risk groups (p=0.749).

Model development

There was a significant non-zero cure fraction for RFS in the training set (p<0.0001) with the estimated percentage of patients cured at 28.5% (Figure 1A). This demonstrates heterogeneity of the outcomes of patients with CN-AML, and that our data are likely composed of two subgroups: one subgroup of patients who are susceptible to the event (relapse or death) and the second of patients who are “cured.”

Prior to applying our penalized semi-parametric MCM, we verified that we had sufficient follow-up data and that a significant non-zero cure fraction was present in our training set. Figure 1A depicts RFS for our training set, estimated using the Kaplan-Meier method. The time at which 95% of events should have occurred was estimated to be 5.3 years and the 95th percentile of observed event times was 4.92 years, whereas the median follow-up among patients alive at their last follow-up was 9.78 years (range, 0.61 to 21.23 years). This, together with the long plateau in the RFS curve, which does not drop down to zero (Figure 1A), provides empirical evidence of sufficient follow-up.

Examination of model predictions in conjunction with known prognostic factors

In the training set, patients predicted to be cured and those predicted to be susceptible with lower risk of relapse or death had lower white blood cell counts (WBC; p=0.02) and percentages of BM blasts (p=0.018) than patients predicted to be susceptible with higher risk of relapse or death (Supplementary Table 5). There were also significant differences among these three groups with respect to *DNMT3A* (p<0.001), *CEPBA*^{bzip} (p=0.035), and *RUNX1* (p=0.036) mutations and the presence of tyrosine kinase domain mutations in the *FLT3* gene (*FLT3*-TKD; p=0.005) and internal tandem duplications of the *FLT3* gene (*FLT3*-ITD; p<0.001), as well in categorization to the 2022 ELN genetic-risk groups (p<0.001) (Supplementary Table 5). However, when we examined the mutational status of genes having mutations that occurred in at least 30 patients, which included *NPM1*, *CEPBA*^{bzip}, *DNMT3A*, *FLT3*-ITD, *FLT3*-TKD, *IDH1*, *IDH2*, *NRAS*, and *PTPN11*, we found significant differences between patients predicted to be cured versus those predicted to be susceptible and between those predicted to be

susceptible with lower and higher risk of relapse or death (Supplementary Figures 3-11). This indicated that our model improved upon inclusion of these mutation data.

Model validation

Patients in our independent test set were diagnosed with AML more recently and therefore their median follow-up of 4.05 years (range, 0.11 to 6.27 years) among patients alive at their last follow-up was shorter than follow-up of patients in our training set (Figure 1D). Thirteen patients underwent an allogeneic HSCT in first CR and were thus censored at the date of HSCT, which also differs from our training set. While the Kaplan-Meier curve does not necessarily show that a cure fraction is present (Figure 1D), because this patient cohort consists of younger CN-AML patients with RNA-seq data available, we considered it a useful proxy test set. In the test set, there were significant differences among patients predicted to be cured versus those predicted to be susceptible with lower and higher risk of relapse or death for *CEBPA*^{bZIP} (p=0.020), *GATA2* (p=0.02), and *WT1* (p=0.017) mutations (Supplementary Table 6).

Biological relevance of genes in our MCM signature

Topp Gene (accessed on August 18, 2023) was used to provide biological insights with respect to genes included in our MCM signature. Several of them were identified in previously published studies. Fourteen genes, namely, *PRTN3*, *SCRN1*, *ARHGEF17*, *PROM1*, *NRXN2*, *SERPING1*, *AK4*, *ITM2C*, *ALDH2*, *FHL1*, *MDFIC*, *CD34*, *ELANE* and *EMP1*, were differentially expressed between AML patients with *NPM1* mutations and those with wild-type *NPM1* (FDR<0.0001) [17], whereas 11 genes,

CDKN2C, *PRR5L*, *PITPNC1*, *FAAH*, *PROM1*, *ITM2C*, *USP13*, *MDFIC*, *RHD*, *GYPA* and *CD34*, were downregulated in AML patients with cytoplasmic *NPM1* localization compared with those without cytoplasmic *NPM1* localization (FDR=0.0001) [18]. We examined the performance of our model in *NPM1*-mutated patients and there was a significant difference in RFS between patients predicted to be cured versus those predicted to be susceptible (p<0.0001) and between those predicted to be susceptible having higher versus lower risk of relapse or death (p<0.0001) (Supplemental Figure 3). Four genes, *IL18*, *PRTN3*, *ALDH2* and *HDC*, were previously identified to be in the *Cebpa* signaling network that was identified from differentially expressed genes in leukemic cells from murine spleens comparing *Cbfb+/MYH11d179-221* and *Cbfb+/MYH11* chimeras (FDR=0.009) [19]. When examining the performance of our model in *CEBPA*^{bZIP}-mutated patients, there was a significant difference in RFS between patients predicted to be cured versus those predicted to be susceptible (p<0.0001) and between those predicted to be susceptible having higher versus lower risk of relapse or death (p<0.0001) (Supplemental Figure 4).

Discussion

Most adult patients diagnosed with acute myeloid leukemia (AML) harbor recurrent chromosomal abnormalities at diagnosis [20,21]. Nevertheless, patients with cytogenetically normal AML (CN-AML) comprise the largest cytogenetic subgroup, ranging from 40% to 49% of all adult patients with AML [22]. Historically, they were considered to have intermediate prognosis [20,21,23]. However, several large studies demonstrated that CN-AML patients are very heterogeneous clinically [23] and

molecularly [1,22,24–32], which has led the European LeukemiaNet (ELN) experts to develop genetic-risk classifications [8,33,34], in which the presence of select gene mutations serves as criteria allowing stratification of CN-AML patients into Favorable, Intermediate, and Adverse genetic-risk groups [8]. Although the prognostic value of these classifications has been validated [35–38], there is still heterogeneity of clinical outcomes among CN-AML patients within the same ELN genetic-risk groups. Thus, there is an unmet clinical need to refine currently used genetic-risk stratification to optimize treatment guidance.

Previous studies found that adult CN-AML patients exhibit more variability in their gene-expression profiles than other commonly observed cytogenetic groups [39–51]. However, some of these studies have included heterogeneous cytogenetic groups in the classifer development [40], included both younger and older CN-AML patients despite the fact that treatment regimens often differ by age group [30–32,40,45,50,51], focused on older CN-AML patients aged ≥60 years [28,29,42–44], or derived gene-expression signatures associated with recurrent gene mutations [1,25–32,43,45,46], or with the expression of selected genes with prognostic significance [42,44,47,48].

In prior studies, development of prognostic models derived using high-throughput gene-expression data selected genes either by assigning patients to two groups, those who remained in CR ≥3 years and those who relapsed, or predicted attainment of CR [49]. Others fit univariate Cox proportional hazards models followed by (1) k-means clustering to identify naturally occurring groups related to time-to-event outcome [40], (2) principal component analysis [50], or (3) a multivariable Cox model [51,52], or validated previously identified gene clustering using compound covariate prediction by

modeling alive versus dead at last follow-up rather than fitting a time-to-event model [41]. However, in several publications, the Kaplan-Meier estimate of survival demonstrates a long plateau that does not drop down to zero despite long follow-up, suggesting the existences of a subgroup of CN-AML patients who enjoy long-term RFS that approaches their population expected survival. In fact, it has been suggested that AML patients attaining 3-year RFS can be considered “potentially cured” [2]. Moreover, in a study of 1,068 AML patients who achieved a CR, the failure rate declined over time, thus the proportional hazards assumption was not satisfied [2]. Our multivariable mixture cure model overcomes problems encountered when the proportional hazards assumption is violated due to the presence of a cured subset and permits modeling a censored time-to-event outcome rather than defining arbitrary groups such as those who remained in CR ≥3 years and those who relapsed.

A recent study sought to identify molecular markers associated with long-term RFS in CN-AML patients by employing a case-control design whereby single-cell RNA sequencing profiles of 28 CN-AML patients remaining in first CR for at least five years were evaluated against 31 well-matched CN-AML patients who relapsed within two years of achieving their first CR [53]. Others developed a prognostic model for patients with CN-AML that was based on the mutational status of select genes (*CEBPA*^{bZIP}, *FLT3*-ITD, *NPM1*) and clinical characteristics (age, WBC, Eastern Cooperative Oncology Group performance status) instead of using high-throughput gene-expression data [54]. They also fit a multivariable Cox PH model, which is known to yield inaccurate estimates when a cure fraction is present.

Therefore, instead of employing a case-control design which requires *a priori* arbitrary timepoints for patient classification, we employed a novel time-to-event model and established a predictive algorithm for identifying long-term survivors using RNA-seq data evaluated in samples collected at the time of diagnosis. Our penalized MCM identified genes associated with cure and latency in younger CN-AML patients, which performed well when applied to an independent test set.

Supplementary references

1. Mendler JH, Maharry K, Radmacher MD, Mrózek K, Becker H, Metzeler KH, et al. *RUNX1* mutations are associated with poor outcome in younger and older patients with cytogenetically normal acute myeloid leukemia and with distinct gene and microRNA expression signatures. J Clin Oncol. 2012;30:3109–18.

2. Yanada M, Garcia-Manero G, Borthakur G, Ravandi F, Kantarjian H, Estey E. Potential cure of acute myeloid leukemia: Analysis of 1069 consecutive patients in first complete remission. Cancer. 2007;110:2756–60.

3. Mrózek K, Carroll AJ, Maharry K, Rao KW, Patil SR, Pettenati MJ, et al. Central review of cytogenetics is necessary for cooperative group correlative and clinical studies of adult acute leukemia: the Cancer and Leukemia Group B experience. Int J Oncol. 2008;33:239–44.

4. Cancer Genome Atlas Research Network, Ley TJ, Miller C, Ding L, Raphael BJ, Mungall AJ, et al. Genomic and epigenomic landscapes of adult de novo acute myeloid leukemia. N Engl J Med. 2013;368:2059–74.

5. Papaemmanuil E, Gerstung M, Bullinger L, Gaidzik VI, Paschka P, Roberts ND, et al. Genomic classification and prognosis in acute myeloid leukemia. N Engl J Med. 2016;374:2209–21.

6. Bamopoulos SA, Batcha AMN, Jurinovic V, Rothenberg-Thurley M, Janke H, Ksienzyk B, et al. Clinical presentation and differential splicing of *SRSF2*, *U2AF1* and *SF3B1* mutations in patients with acute myeloid leukemia. Leukemia. 2020;34:2621–34.

7. Braess J, Spiekermann K, Staib P, Grüneisen A, Wörmann B, Ludwig W-D, et al. Dose-dense induction with sequential high-dose cytarabine and mitoxantone (S-HAM) and pegfilgrastim results in a high efficacy and a short duration of critical neutropenia in de novo acute myeloid leukemia: a pilot study of the AMLCG. Blood. 2009;113:3903–10.

8. Döhner H, Wei AH, Appelbaum FR, Craddock C, DiNardo CD, Dombret H, et al. Diagnosis and management of AML in adults: 2022 ELN recommendations from an international expert panel. Blood. 2022;140:1345–77.

9. Laska EM, Meisner MJ. Nonparametric estimation and testing in a cure model. Biometrics. 1992;48:1223.

10. Othus M, Bansal A, Erba H, Ramsey S. Bias in mean survival from fitting cure models with limited follow-up. Value Health. 2020;23:1034–9.

11. Goldman AI. Survivorship analysis when cure is a possibility: A Monte Carlo study. Stat Med. 1984;3:153–63.

12. Maller RA, Zhou X. Survival Analysis with Long-Term Survivors. Chichester ; New York: Wiley; 1996.

13. Kuk AYC, Chen C-H. A mixture model combining logistic regression with proportional hazards regression. Biometrika. 1992;79:531–41.

14. Dempster AP, Laird NM, Rubin DB. Maximum likelihood from incomplete data via the EM algorithm. J R Stat Soc Series B Stat Methodol. 1977;39:1–38.

15. Law CW, Chen Y, Shi W, Smyth GK. voom: precision weights unlock linear model analysis tools for RNA-seq read counts. Genome Biol. 2014;15:R29.

16. Johnson WE, Li C, Rabinovic A. Adjusting batch effects in microarray expression data using empirical Bayes methods. Biostatistics. 2007;8:118–27.

17. Verhaak RGW, Goudswaard CS, van Putten W, Bijl MA, Sanders MA, Hugens W, et al. Mutations in nucleophosmin (*NPM1*) in acute myeloid leukemia (AML): association with other gene abnormalities and previously established gene expression signatures and their favorable prognostic significance. Blood. 2005;106:3747–54.

18. Alcalay M, Tiacci E, Bergomas R, Bigerna B, Venturini E, Minardi SP, et al. Acute myeloid leukemia bearing cytoplasmic nucleophosmin (NPMc+ AML) shows a distinct gene expression profile characterized by up-regulation of genes involved in stem-cell maintenance. Blood. 2005;106:899–902.

19. Kamikubo Y, Zhao L, Wunderlich M, Corpora T, Hyde RK, Paul TA, et al. Accelerated leukemogenesis by truncated CBFβ-SMMHC defective in high-affinity binding with RUNX1. Cancer Cell. 2010;17:455–68.

20. Grimwade D, Walker H, Oliver F, Wheatley K, Harrison C, Harrison G, et al. The importance of diagnostic cytogenetics on outcome in AML: analysis of 1,612 patients entered into the MRC AML 10 trial. Blood. 1998;92:2322–33.

21. Byrd JC, Mrózek K, Dodge RK, Carroll AJ, Edwards CG, Arthur DC, et al. Pretreatment cytogenetic abnormalities are predictive of induction success, cumulative

incidence of relapse, and overall survival in adult patients with de novo acute myeloid leukemia: results from Cancer and Leukemia Group B (CALGB 8461). Blood. 2002;100:4325–36.

22. Mrózek K, Marcucci G, Paschka P, Whitman SP, Bloomfield CD. Clinical relevance of mutations and gene-expression changes in adult acute myeloid leukemia with normal cytogenetics: are we ready for a prognostically prioritized molecular classification? Blood. 2007;109:431–48.

23. Farag SS, Ruppert AS, Mrózek K, Mayer RJ, Stone RM, Carroll AJ, et al. Outcome of induction and postremission therapy in younger adults with acute myeloid leukemia with normal karyotype: a Cancer and Leukemia Group B study. J Clin Oncol. 2005;23:482–93.

24. Marcucci G, Mrózek K, Bloomfield CD. Molecular heterogeneity and prognostic biomarkers in adults with acute myeloid leukemia and normal cytogenetics. Curr Opin Hematol. 2005;12:68–75.

25. Marcucci G, Maharry K, Radmacher MD, Mrózek K, Vukosavljevic T, Paschka P, et al. Prognostic significance of, and gene and microRNA expression signatures associated with, *CEBPA* mutations in cytogenetically normal acute myeloid leukemia with high-risk molecular features: A Cancer and Leukemia Group B study. J Clin Oncol. 2008;26:5078–87.

26. Taskesen E, Bullinger L, Corbacioglu A, Sanders MA, Erpelinck CAJ, Wouters BJ, et al. Prognostic impact, concurrent genetic mutations, and gene expression features of AML with *CEBPA* mutations in a cohort of 1182 cytogenetically normal AML patients: further evidence for *CEBPA* double mutant AML as a distinctive disease entity. Blood. 2011;117:2469–75.

27. Whitman SP, Ruppert AS, Radmacher MD, Mrózek K, Paschka P, Langer C, et al. *FLT3* D835/I836 mutations are associated with poor disease-free survival and a distinct gene-expression signature among younger adults with de novo cytogenetically normal acute myeloid leukemia lacking *FLT3* internal tandem duplications. Blood. 2008;111:1552–9.

28. Becker H, Marcucci G, Maharry K, Radmacher MD, Mrózek K, Margeson D, et al. Mutations of the Wilms tumor 1 gene (*WT1*) in older patients with primary cytogenetically normal acute myeloid leukemia: a Cancer and Leukemia Group B study. Blood. 2010;116:788–92.

29. Becker H, Marcucci G, Maharry K, Radmacher MD, Mrózek K, Margeson D, et al. Favorable prognostic impact of *NPM1* mutations in older patients with cytogenetically normal de novo acute myeloid leukemia and associated gene- and microRNA-expression signatures: A Cancer and Leukemia Group B study. J Clin Oncol. 2010;28:596–604.

30. Marcucci G, Maharry K, Wu Y-Z, Radmacher MD, Mrózek K, Margeson D, et al. *IDH1* and *IDH2* gene mutations identify novel molecular subsets within de novo cytogenetically normal acute myeloid leukemia: a Cancer and Leukemia Group B study. J Clin Oncol. 2010;28:2348–55.

31. Greif PA, Konstandin NP, Metzeler KH, Herold T, Pasalic Z, Ksienzyk B, et al. *RUNX1* mutations in cytogenetically normal acute myeloid leukemia are associated with a poor prognosis and up-regulation of lymphoid genes. Haematologica. 2012;97:1909–15.

32. Metzeler KH, Maharry K, Radmacher MD, Mrózek K, Margeson D, Becker H, et al. *TET2* mutations improve the new European LeukemiaNet risk classification of acute myeloid leukemia: a Cancer and Leukemia Group B study. J Clin Oncol. 2011;29:1373–81.

33. Döhner H, Estey EH, Amadori S, Appelbaum FR, Büchner T, Burnett AK, et al. Diagnosis and management of acute myeloid leukemia in adults: recommendations from an international expert panel, on behalf of the European LeukemiaNet. Blood. 2010;115:453–74.

34. Döhner H, Estey E, Grimwade D, Amadori S, Appelbaum FR, Büchner T, et al. Diagnosis and management of AML in adults: 2017 ELN recommendations from an international expert panel. Blood. 2017;129:424–47.

35. Mrózek K, Marcucci G, Nicolet D, Maharry KS, Becker H, Whitman SP, et al. Prognostic significance of the European LeukemiaNet standardized system for reporting cytogenetic and molecular alterations in adults with acute myeloid leukemia. J Clin Oncol. 2012;30:4515–23.

36. Herold T, Rothenberg-Thurley M, Grunwald VV, Janke H, Goerlich D, Sauerland MC, et al. Validation and refinement of the revised 2017 European LeukemiaNet genetic risk stratification of acute myeloid leukemia. Leukemia. 2020;34:3161–72.

37. Eisfeld A-K, Kohlschmidt J, Mims A, Nicolet D, Walker CJ, Blachly JS, et al. Additional gene mutations may refine the 2017 European LeukemiaNet classification in adult patients with de novo acute myeloid leukemia aged <60 years. Leukemia. 2020;34:3215–27.

38. Mrózek K, Kohlschmidt J, Blachly JS, Nicolet D, Carroll AJ, Archer KJ, et al. Outcome prediction by the 2022 European LeukemiaNet genetic-risk classification for adults with acute myeloid leukemia: an Alliance study. Leukemia. 2023;37:788–98.

39. Debernardi S, Lillington DM, Chaplin T, Tomlinson S, Amess J, Rohatiner A, et al. Genome-wide analysis of acute myeloid leukemia with normal karyotype reveals a unique pattern of homeobox gene expression distinct from those with translocation-mediated fusion events. Genes Chromosomes Cancer. 2003;37:149–58.

40. Bullinger L, Döhner K, Bair E, Fröhling S, Schlenk RF, Tibshirani R, et al. Use of gene-expression profiling to identify prognostic subclasses in adult acute myeloid leukemia. N Engl J Med. 2004;350:1605–16.

41. Radmacher MD, Marcucci G, Ruppert AS, Mrózek K, Whitman SP, Vardiman JW, et al. Independent confirmation of a prognostic gene-expression signature in adult acute myeloid leukemia with a normal karyotype: a Cancer and Leukemia Group B study. Blood. 2006;108:1677–83.

42. Schwind S, Marcucci G, Maharry K, Radmacher MD, Mrózek K, Holland KB, et al. *BAALC* and *ERG* expression levels are associated with outcome and distinct gene and microRNA expression profiles in older patients with de novo cytogenetically normal acute myeloid leukemia: a Cancer and Leukemia Group B study. Blood. 2010;116:5660–9.

43. Schwind S, Marcucci G, Kohlschmidt J, Radmacher MD, Mrózek K, Maharry K, et al. Low expression of *MN1* associates with better treatment response in older patients with de novo cytogenetically normal acute myeloid leukemia. Blood. 2011;118:4188–98.

44. Metzeler KH, Heilmeier B, Edmaier KE, Rawat VPS, Dufour A, Döhner K, et al. High expression of lymphoid enhancer-binding factor-1 (*LEF1*) is a novel favorable prognostic factor in cytogenetically normal acute myeloid leukemia. Blood. 2012;120:2118–26.

45. Becker H, Maharry K, Radmacher MD, Mrózek K, Metzeler KH, Whitman SP, et al. Clinical outcome and gene- and microRNA-expression profiling according to the Wilms tumor 1 (*WT1*) single nucleotide polymorphism rs16754 in adult de novo cytogenetically normal acute myeloid leukemia: a Cancer and Leukemia Group B study. Haematologica. 2011;96:1488–95.

46. Bullinger L, Döhner K, Kranz R, Stirner C, Fröhling S, Scholl C, et al. An FLT3 gene-expression signature predicts clinical outcome in normal karyotype AML. Blood. 2008;111:4490–5.

47. Langer C, Radmacher MD, Ruppert AS, Whitman SP, Paschka P, Mrózek K, et al. High *BAALC* expression associates with other molecular prognostic markers, poor outcome, and a distinct gene-expression signature in cytogenetically normal patients younger than 60 years with acute myeloid leukemia: a Cancer and Leukemia Group B (CALGB) study. Blood. 2008;111:5371–9.

48. Langer C, Marcucci G, Holland KB, Radmacher MD, Maharry K, Paschka P, et al. Prognostic importance of *MN1* transcript levels, and biologic insights from *MN1*-associated gene and microRNA expression signatures in cytogenetically normal acute myeloid leukemia: a Cancer and Leukemia Group B study. J Clin Oncol. 2009;27:3198–204.

49. Walker CJ, Mrózek K, Ozer HG, Nicolet D, Kohlschmidt J, Papaioannou D, et al. Gene expression signature predicts relapse in adult patients with cytogenetically normal acute myeloid leukemia. Blood Adv. 2021;5:1474–82.

50. Metzeler KH, Hummel M, Bloomfield CD, Spiekermann K, Braess J, Sauerland M-C, et al. An 86-probe-set gene-expression signature predicts survival in cytogenetically normal acute myeloid leukemia. Blood. 2008;112:4193–201.

51. Yang L, Zhang H, Yang X, Lu T, Ma S, Cheng H, et al. Prognostic prediction of cytogenetically normal acute myeloid leukemia based on a gene expression model. Front Oncol. 2021;11:659201.

52. Huang R, Liao X, Li Q. Identification and validation of potential prognostic gene biomarkers for predicting survival in patients with acute myeloid leukemia. Onco Targets Ther. 2017;10:5243–54.

53. Ferraro F, Miller CA, Christensen KA, Helton NM, O’Laughlin M, Fronick CC, et al. Immunosuppression and outcomes in adult patients with de novo acute myeloid leukemia with normal karyotypes. Proc Natl Acad Sci USA. 2021;118:e2116427118.

54. Pastore F, Dufour A, Benthaus T, Metzeler KH, Maharry KS, Schneider S, et al. Combined molecular and clinical prognostic index for relapse and survival in cytogenetically normal acute myeloid leukemia. J Clin Oncol. 2014;32:1586–94.
